# Quantum rectangular MinRank attack on multi-layer UOV signature schemes

**DOI:** 10.1038/s41598-024-66841-0

**Published:** 2024-07-16

**Authors:** Seong-Min Cho, Seung-Hyun Seo

**Affiliations:** 1 Department of Electrical Engineering, Graduate School of Hanyang University, Seoul, 04763 South Korea; 2 Division of Electrical Engineering, Hanyang University ERICA, Ansan, 15588 South Korea

**Keywords:** Quantum information, Information technology

## Abstract

Recent rank-based attacks have reduced the security of Rainbow, which is one of the multi-layer UOV signatures, below the NIST security requirements by speeding up iterative kernel-finding operations using classical mathematics techniques. If quantum algorithms are applied to perform these iterative operations, the rank-based attacks may be more threatening to multi-layer UOV, including Rainbow. In this paper, we propose a quantum rectangular MinRank attack called the Q-rMinRank attack, the first quantum approach to key recovery attacks on multi-layer UOV signatures. Our attack is a general model applicable to multi-layer UOV signature schemes, and in this paper, we provide examples of its application to Rainbow and the Korean TTA standard, HiMQ. We design two quantum oracle circuits to find the kernel in consideration of the depth-width trade-off of quantum circuits. One is to reduce the width of the quantum circuits using qubits as a minimum, and the other is to reduce the depth using parallelization instead of using a lot of qubits. By designing quantum circuits to find kernels with fewer quantum resources and complexity by adding mathematical techniques, we achieve quadratic speedup for the MinRank attack to recover the private keys of multi-layer UOV signatures. We also estimate quantum resources for the designed quantum circuits and analyze quantum complexity based on them. The width-optimized circuit recovers the private keys of Rainbow parameter set V with only 1089 logical qubits. The depth-optimized circuit recovers the private keys of Rainbow parameter set V with a quantum complexity of $2^{174}$, which is lower than the complexity of $2^{221}$ recovering the secret key of AES-192, which provides the same security level as parameter set III.

## Introduction

Due to recent advances in the development of quantum computers, such as Google’s 53-qubit quantum processor “Sycamore”^1^ and IBM’s 127-qubit quantum processor “Eagle”^2^, the National Institute of Standards and Technology (NIST) estimates that quantum computers will be capable of breaking 2048-bit RSA as early as 2026. This is because 4096 qubits in a quantum computing environment using Shor’s quantum algorithm^3^ are enough to break 2048-bit RSA^4^. The possibility that current public-key cryptographic algorithms such as RSA and ECDSA will be broken in a quantum computing environment has led NIST to conduct a Post-Quantum Cryptography (PQC) standardization project. To be securely used in quantum computing environments, the PQC candidates must meet the security requirements set out by NIST, from 128-bit security for Level I to 256-bit security for Level V.

Among the PQC candidates for the NIST standardization project, Multivariate Quadratic (MQ)-based signature schemes are expected to be highly utilized in IoT devices with limited resources due to the advantages of short signature length as well as fast signature generation and verification speed^5^. One of the prominent MQ-based signature schemes widely studied is the Unbalanced Oil and Vinegar (UOV) signature, known for its simplicity and security. Since the UOV signature is constructed as a single-layer structure, which generates signatures by finding solutions for all oil variables in the MQ system at once, the signature generation is somewhat slow. To improve performance speeds, multi-layer UOV signature schemes have been proposed, where the oil variables of the MQ system are divided into layers. The multi-layer UOV signatures improve the signature generation speed by finding solutions for fewer oil variables in each layer. One such scheme is Rainbow, proposed by Jintai Ding^6^. It had been considered one of the NIST PQC standardization finalists until recently. In Korea, HiMQ, a multi-layer UOV signature, was standardized by the Korean Telecommunications Technology Association (TTA) in 2020^7^. HiMQ utilizes a sparse central map to achieve a small private key size and fast signature generation.

The security of prominent MQ-based signatures, such as UOV, mainly relies on the hardness of the MQ problem and the Extended Isomorphism of Polynomials (EIP) problem. Among them, multi-layer UOV signatures require the additional hardness of the MinRank problem to ensure security. The existential unforgeability of multi-layer UOV signatures is based on the intractability of the MQ problem (solving multivariate systems of quadratic equations). The difficulty of recovering private keys from the public keys of multi-layer UOV signatures depends on the difficulties of the EIP and the MinRank (finding a non-zero *k*-tuple such that the rank of a linear combination of some matrices is less than some small rank) problems. Thus, signature forgery attacks and key recovery attacks on multi-layer UOV signatures have attempted to solve these underlying problems.

So far, there have been several attempts to forge multi-layer UOV signatures, especially the Rainbow signature scheme, by trying to solve the MQ problem using mathematical techniques such as XL^8^ and Gr$\mathrm {\ddot{o}}$bner Basis algorithms^9^, but they have not been successful. Since Olivier Billet et al. proposed a MinRank attack algorithm to recover private keys from the public keys of Rainbow in 2006^10^, key recovery attacks such as the MinRank attack have become a potential threat to Rainbow. In 2020, Bardet et al. reduced the complexity of the MinRank attacks on Rainbow to $\frac{1}{3}$ of the existing MinRank attacks in parameter sets III and V of Rainbow^11^. However, the attack proposed by Bardet et al. did not even threaten the parameter sets submitted for the second round of the NIST PQC project.

In 2021, Ward Beullens proposed a rectangular MinRank attack^12^. He converted the public keys of Rainbow to polar forms and reduced the size of the input matrices of the MinRank attack using the polar forms. Then, he recovered the private keys of Rainbow using the Support Minors Modeling algorithm^11^. This attack reduced the security level of parameter sets I, III, and V of Rainbow to 127-bit, 177-bit, and 226-bit security levels, respectively. In 2022, Ward Beullens proposed two new key recovery attacks (a simple attack and a combined attack) against Rainbow, further reducing the security level of Rainbow^13^. He reduced the complexity of the MinRank attack by suggesting a method to guess the kernel with a high probability in a simple attack. They also reduced the attack complexity for parameter sets III and V by combining a rectangular MinRank attack^12^ with a simple attack. Moreover, he reduced the security level of parameter sets I, III, and V of Rainbow to 69-bit, 160-bit, and 257-bit security levels in their simple attack and 99-bit, 157-bit, and 206-bit security levels in their combined attack, respectively. Parameter set I of Rainbow does not fall short of the minimum security requirement, the 128-bit security level, against such key recovery attacks. However, parameter sets III and V of Rainbow still satisfy the 128-bit (I) and 196-bit (III) security levels set by NIST, even against the most threatening key recovery attack proposed by Ward Beullens^12,13^.

The cost of iterative operations to find a kernel dominates the complexity of rank-based attacks against multi-layer UOV signatures. Until now, classical key recovery attacks^10–13^ have improved attack complexity by using mathematical techniques such as the Support Minors Modeling algorithm^11^ and the Wiedemann algorithm^14^ to find the kernel quickly. If quantum properties such as superposition to find the kernel are utilized, we could achieve speed-ups in kernel search. The Rainbow team evaluated the security level of Rainbow against Grover’s algorithm-based quantum attacks by simple numerical calculation ($O(N) \rightarrow O(\sqrt{N})$). They did not show any concrete oracle circuits for the quantum attacks. For accurate quantum complexity estimation, it is necessary to present Grover oracle quantum circuits and estimate their quantum resources.

We present complete Grover oracle circuits, called Q-rMinRank-Grover, for quantum key recovery attacks on multi-layer UOV signatures for the first time. In contrast to the Rainbow team’s quantum analysis approach, we obtained precise and reliable estimates of the quantum resources required to compute these kernel searching operations using our Grover oracle circuits, called Q-rMinRank_Oracle. We achieved speed-ups in kernel search by combining quantum properties and classical techniques for the MinRank attack. Moreover, we reduced the size of the quantum search domain through the polar form conversion of the public key matrix to rectangular forms.

To design the quantum circuit, Q-rMinRank_Oracles, we reflect the depth-width trade-off considering the Quantum Volume (QV). The QV is a benchmark measure to quantify the capability of Noisy Intermediate-Scale Quantum (NISQ) devices. The QV measures the performance of a quantum circuit by considering the width and depth of a quantum circuit^15^. The width of a quantum circuit represents the number of qubits used, and the depth describes the number of layers composed of quantum gates that are executed simultaneously in parallel. A quantum circuit with a small width means using less memory on a quantum computer, and a short depth ensures a fast running time. The depth of a quantum circuit is essential for NIST’s quantum complexity analysis. NIST defines metrics reflecting a variety of predictions about the development of quantum computing technology to evaluate security against such quantum attacks^16^. The metrics are based on an approach that restricts quantum attacks to a fixed running time or circuit depth. Therefore, we design the Q-rMinRank_Oracles in two ways considering these depth-width trade-offs. The first is a depth-optimized oracle $\mathcal {O}_1$, and the second is a width-optimized oracle $\mathcal {O}_2$. In environments where fast running time is important, the quantum circuit can be constructed with $\mathcal {O}_1$. In environments where the number of available qubits is small, the circuit can be constructed with $\mathcal {O}_2$.

We also estimate the quantum resources of the Q-rMinRank-Grover. The quantum circuits consisting of $\mathcal {O}_1$ require 1097, 5757, and 8057 logical qubits for key recovery of Rainbow’s parameter sets I, III, and V, respectively, and 3662, 6459, and 7036 logical qubits for key recovery of HiMQ’s parameter sets HiMQ-128, HiMQ-160, and HiMQ-192, respectively. And the quantum circuits consisting of $\mathcal {O}_2$ require 289, 833, and 1089 logical qubits for key recovery of Rainbow’s parameter sets I, III, and V, respectively, and 665, 1121, and 1312 logical qubits for key recovery of HiMQ’s parameter sets HiMQ-128, HiMQ-160, and HiMQ-192, respectively. Based on the quantum resource estimations, we analyze the complexity of the Q-rMinRank-Grover based on NIST’s quantum complexity estimation. In the submission requirements document for the PQC standardization project in 2016^16^, NIST specified the quantum complexity criteria that PQCs must meet, based on the quantum resource estimates of Grover’s algorithm for AES key recovery by M. Grassl et al^17^. This criterion was revised in 2022 for the additional digital signature standardization project^18^ to reflect the quantum resource estimates of the improved Grover’s algorithm by S. Jaques et al. in 2020^19^. NIST notes that the quantum complexities of $2^{157}$, $2^{221}$, and $2^{285}$ correspond to PQC security levels I, III, and V, respectively. The quantum complexity for key recovery of Rainbow parameter set V is $1.80\times 2^{174}$, which is lower than the complexity of quantum key search attacks on AES-192 presented by NIST ($2^{221}$), and the quantum complexity for parameter set III is $1.01\times 2^{158}$, which is close to the quantum complexity for AES-128 ($2^{157}$). Inevitably, the quantum complexity for parameter set I ($1.90\times 2^{93}$) is far below the quantum complexity for AES-128. While the existing classical attacks on Rainbow only succeed up to parameter set I (reducing the security level of parameter set III from 192 to 157, but still considerably larger than 128), our Q-rMinRank attacks can succeed up to parameter set III if more efficient quantum arithmetic circuits are available in the future. For the key recovery of HiMQ, the quantum complexity of our Q-rMinRank attack on parameter set HiMQ-128 is around $1.48\times 2^{113}$, which does not satisfy NIST’s security level I. The remaining parameter sets (HiMQ-160 and HiMQ-192) do not satisfy NIST’s security level III. To verify the feasibility of our oracle, we implemented the quantum circuit designed for a toy example of Rainbow with the quantum simulators Qiskit^20^ and ProjectQ^21^.

The remaining paper is organized as follows: The “Preliminaries” section provides preliminary information of the elementary quantum gates, the Rainbow signature scheme, the MinRank attack, and Grover’s algorithm^22^. In the “Quantum rectangular MinRank attack” section, we present a quantum rectangular MinRank attack. The design methods of oracle circuits for the quantum rectangular MinRank attack are shown in the “Oracle circuit designs” section. In the ”Complexity analysis” section, we evaluate the security level of the Rainbow scheme against the Q-rMinRank attack and estimate the quantum resources required for the Q-rMinRank-Grover and quantum complexities. Finally, we show the quantum simulation result in the “Quantum simulation result” section. For the sake of convenience in the explanation, in the “Quantum rectangular MinRank attack” and “Oracle circuit designs” sections, we describe our attack method based on Rainbow. Our attack and analysis of HiMQ are detailed in the Supplementary Material as an appendix.

## Preliminaries

We introduce the elementary quantum gates of quantum circuits, the Rainbow signature scheme, and Grover’s quantum algorithm in this section.

### Elementary quantum gates

#### NOT gate

**Figure 1:**
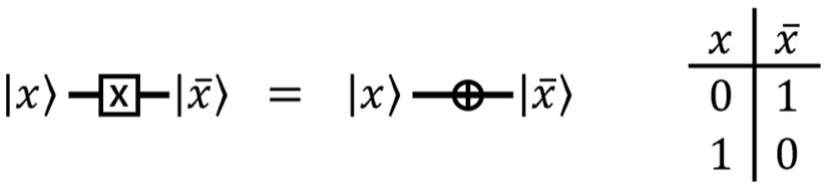
The quantum $\textsf{NOT}$ gate.

The quantum $\textsf{NOT}$ gate is also called a $\textsf{X}$ gate. The quantum $\textsf{NOT}$ gate flips the quantum state ${|{0}\rangle }$ to ${|{1}\rangle }$ and ${|{1}\rangle }$ to ${|{0}\rangle }$ as shown in Fig. 1.

#### CNOT gate

**Figure 2:**
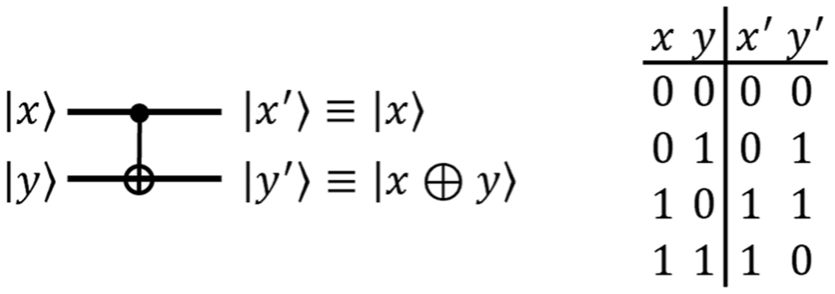
The quantum $\textsf{CNOT}$ gate.

The quantum $\textsf{controlled}$-$\textsf{NOT}$ ($\textsf{CNOT}$) gate takes in two qubits, ${|{x}\rangle }$ and ${|{y}\rangle }$, and outputs ${|{x}\rangle }$ and ${|{x\oplus y}\rangle }$ as shown in Fig. 2. In quantum circuits, $\textsf{CNOT}$ gates are used for XOR (addition over modulo 2) operations.

#### C^N^-NOT gate

**Figure 3:**
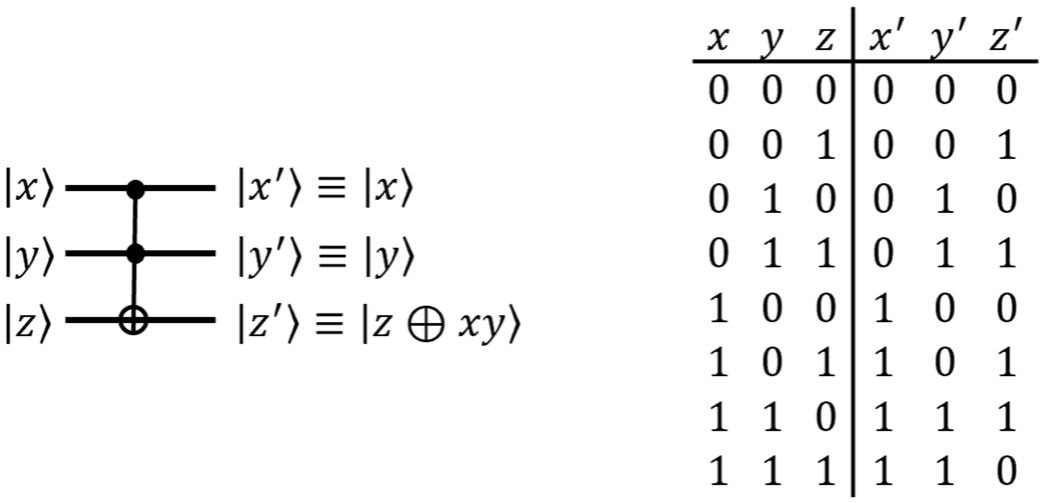
The quantum $\textsf{CCNOT}$ gate.

**Figure 4:**
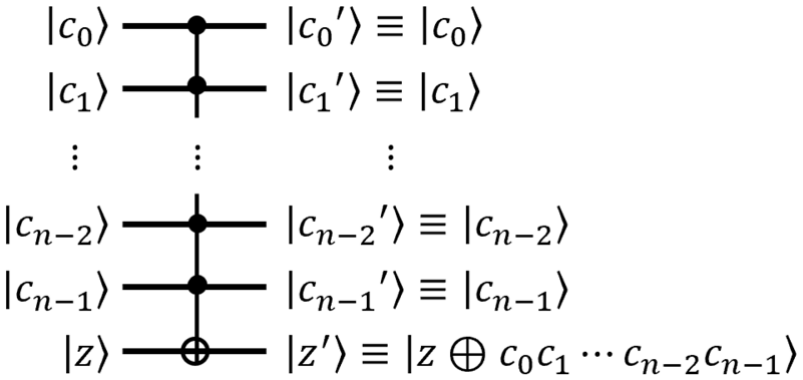
The quantum $\textsf{C}^N$-$\textsf{NOT}$ gate.

**Figure 5:**
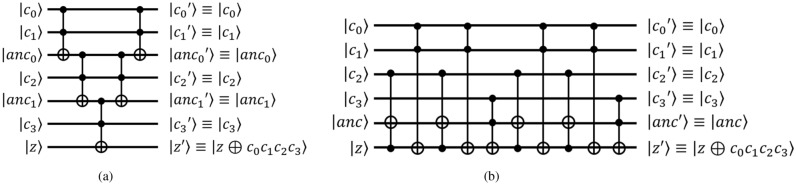
The examples of $\textsf{C}^N$-$\textsf{NOT}$ decomposition ($N=4$).

- $\textsf{CCNOT}$ Gate The quantum $\textsf{controlled}$-$\textsf{controlled}$-$\textsf{NOT}$ ($\textsf{CCNOT}$) gate, also called a $\textsf{Toffoli}$ gate, has two control qubits and one target qubit as inputs. The $\textsf{Toffoli}$ gate flips the target qubit ${|{z}\rangle }$ only when the two control qubits ${|{x}\rangle }$ and ${|{y}\rangle }$ are both in state ${|{1}\rangle }$, i.e. the state of the target qubit is ${|{z\oplus xy}\rangle }$. $\textsf{Toffoli}$ gates are often used in quantum circuits for bitwise multiplication Fig. 3.
- $\textsf{C}^N$-$\textsf{NOT}$ Gate The $\textsf{C}^N$-$\textsf{NOT}$ gate in Fig. 4 reverses qubit *z* when *N* control qubits $c_i$ are all 1. That is, when $N+1$ qubits $c_0$, $c_1$, ... , $c_{n-1}$, and *z* are input to this $\textsf{C}^N$-$\textsf{NOT}$ gate, the result of this $\textsf{C}^N$-$\textsf{NOT}$ gate is $c_0$, $c_1$, ... , $c_{n-1}$, and $z'=z\oplus c_0c_1...c_{n-1}$. The $\textsf{C}^N$-$\textsf{NOT}$ gate consists of $\textsf{CCNOT}$ gates, and there are two methods to implement the $\textsf{C}^N$-$\textsf{NOT}$ gate^23^. The first implementation method, named $\textsf{C}^N-\textsf{NOT}_\textsf{D}$, has a small depth but many ancilla qubits, while the second implementation method, named $\textsf{C}^N$-$\textsf{NOT}_\textsf{W}$, has a large depth but only one ancilla qubit. Figure 5a and b show the circuits of $\textsf{C}^N$-$\textsf{NOT}_\textsf{D}$ and $\textsf{C}^N$-$\textsf{NOT}_\textsf{W}$ when $N=4$, respectively. Table 1 shows the number of $\textsf{Toffoli}$ gates, depth, and ancillary qubits required to implement the $\textsf{C}^N$-$\textsf{NOT}$ gate.

**Table 1:** $\textsf{Toffoli}$ resources to impelment $\textsf{C}^N$-$\textsf{NOT}$ gate combining $\textsf{Toffoli}$ gates.

Implementation methods	#Toffoli	#TD	#AQB
$\textsf{C}^4$-$\textsf{NOT}_\textsf{D}$	$2N-3$	$2N-3$	$N-2$
$\textsf{C}^4$-$\textsf{NOT}_\textsf{W}$	$8N-24$	$8N-24$	1

#Toffoli: The number of Toffoli gates

#TD: The depth of Toffoli gates

#AQB: The number of ancilla qubits.

#### Quantum adder and multiplier over $\mathbb {F}_q$

**Figure 6:**
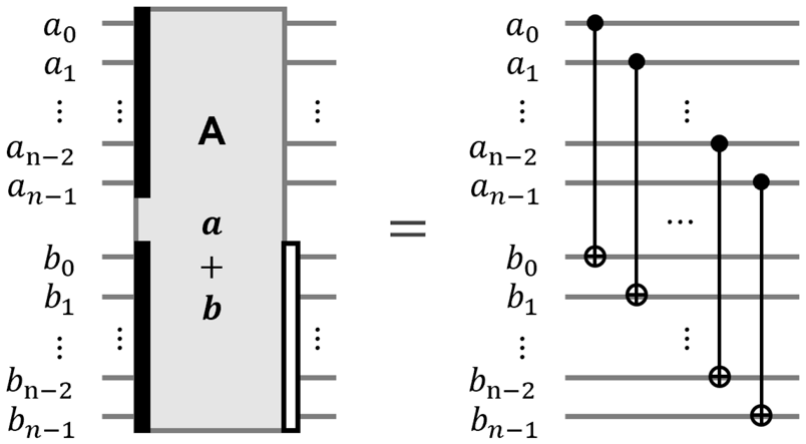
The quantum adder over $\mathbb {F}_{2^n}$ ($\textsf{ADD}$ gate).

The addition on the finite field consists of bitwise XOR operations. So, the quantum adder over $\mathbb {F}_q$, named $\textsf{ADD}$, is simply implemented with only the $\textsf{CNOT}$ gates, as shown in Fig. 6. The quantum adder over $\mathbb {F}_{2^n}$ requires *n* $\textsf{CNOT}$ gates with depth one.

**Table 2:** Quantum resources of quantum multipliers $(n_q = \log _2{q})$.

Quantum multiplier	$n_q$	Depth	# of Gates		# of Ancilla qubits
			$\textsf{NOT}$ gates	$\textsf{CNOT}$ gates	
^24^	4	32	0	160	12
	8	96	0	640	24
^25^	4	17	88	9	15
	8	23	300	27	57

**Figure 7:**
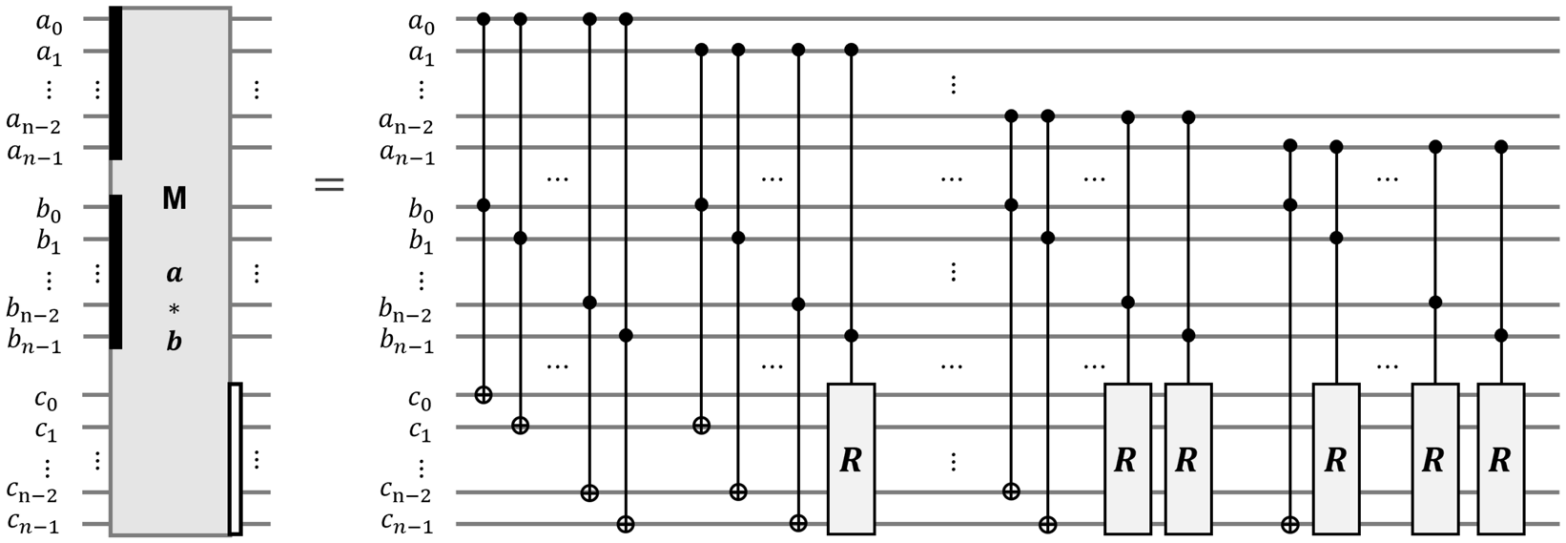
The quantum multiplier over $\mathbb {F}_{2^n}$ ($\textsf{MULT}$ gate) proposed by Cho et al.^24^.

**Figure 8:**
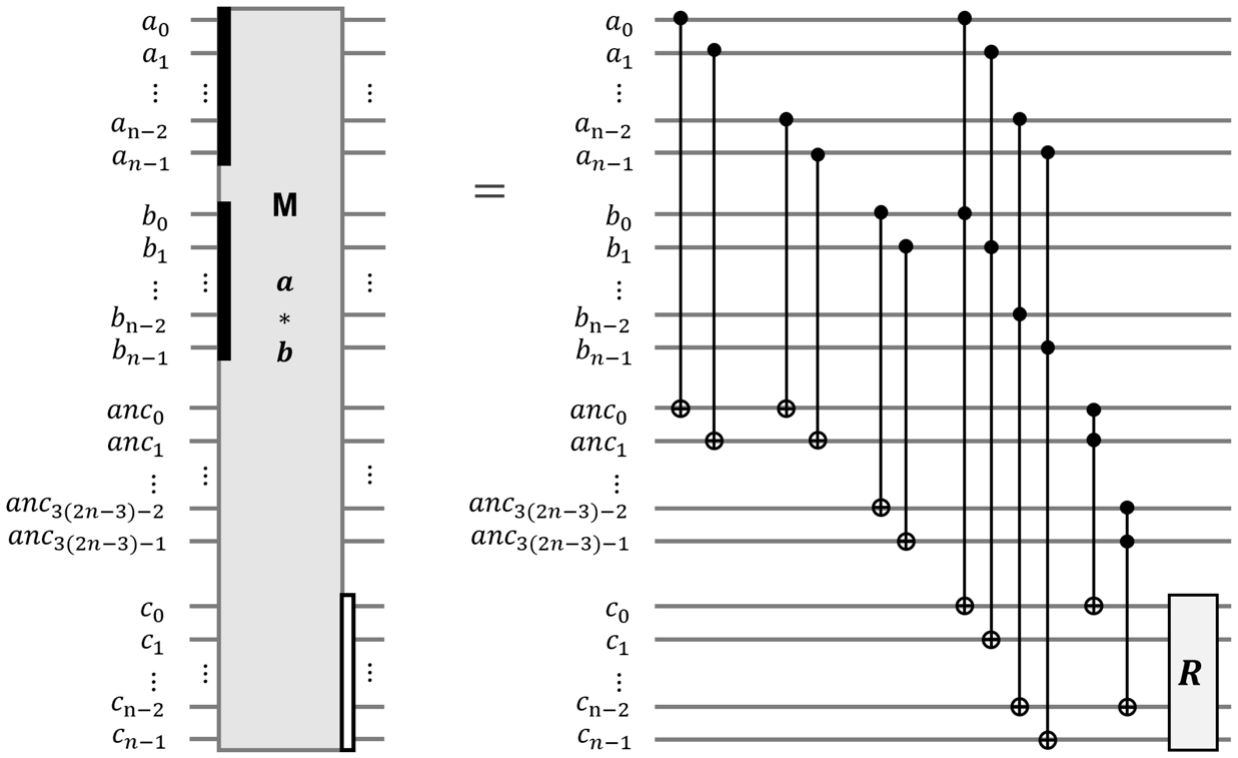
The quantum multiplier over $\mathbb {F}_{2^n}$ ($\textsf{MULT}$ gate) proposed by Jang et al.^25^.

Since multiplication requires a reduction step, how efficiently reduction is calculated when implementing it as a quantum circuit is important. In 2020, Cho et al. proposed efficient quantum multipliers over $\mathbb {F}_{2^n}$ and $\mathbb {F}_{2^{n-1}}$, as shown in Fig. 7^24^. They reduced the number of quantum gates and depth by half. In 2022, Jang et al. proposed quantum multipliers with Toffoli depth one, as shown in Fig. 8^25^. They iterated the Karatsuba multiplication method and reduced the $\textsf{Toffoli}$ depth to one by adding ancilla qubits. A quantum-classical version of the multiplier of Jang et al.^25^ has depth one of the $\textsf{CNOT}$ gate. Table 2 shows the quantum resources for two quantum multipliers^24,25^.

### Rainbow signature scheme

Rainbow, proposed by Jintai Ding^6^, was the only MQ-based cryptography candidate for the third round of the NIST Post Quantum Cryptography (PQC) standardization project. Rainbow’s layered structure has a relatively short signature length compared to other NIST PQC standardization candidates and enables efficient signature generation and verification. In this section, we describe the key generation, signature generation, and signature verification algorithms of Rainbow.

**Figure 9:**
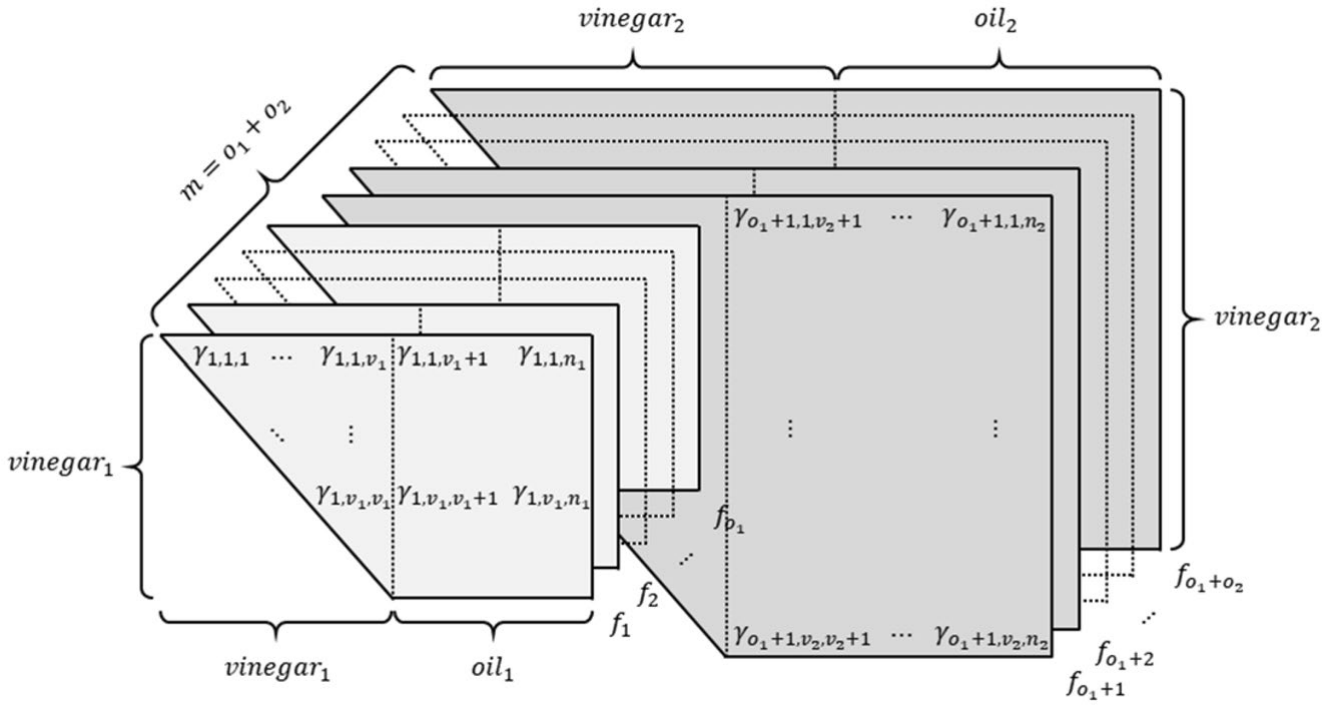
The configuration of the central map *F*.

- *Parameters*
  - a finite field with *q* elements, $\mathbb {F}_q$
  - index sets $V_i =\{1,...,v_i\}, O_i =\{v_i+1,...,v_{i+1}\}$ ($i=1,2$). Note that each $k \in v_{1}+1,..., n$ is contained in exactly one of the sets $O_i$.
  - we have $\Vert V_i\Vert =v_i$ and set $o_i=\Vert O_i\Vert$ ($i=1,2$)
  - the number of equations: $m=n-v_1$ ($m=o_1+o_2$)
  - the number of variables: $n=v_1+o_1+o_2$
- *Key generation*
  - Private Key The private key of Rainbow consists of two affine maps $S: \mathbb {F}^{m}_q \rightarrow \mathbb {F}^{m}_q$ and $T: \mathbb {F}^{n}_q \rightarrow \mathbb {F}^{n}_q$, and the central map $F: \mathbb {F}^{n}_q \rightarrow \mathbb {F}^{m}_q$. The central map *F* consists of two layers, as shown in Fig. 9. The central map of the first layer consists of $o_1$ multivariate equations $f^{(v_{1}+1)}, ..., f^{(v_1+o_1)}$, and the central map of the second layer consists of $o_2$ multivariate equations $f^{(v_{1}+o_1+1)},..., f^{(v_1+n)}$. When $k \in (v_{1}+1, ..., n)$ and *l* is the number of layers, $f^{(k)}$ is as follows:
    1 $$\begin{aligned} f^{(k)}(x_{1},..., x_{n})= \sum _{i, j \in V_{l}}^{i \le j}{\alpha _{ij}^{(k)}x_{i}x_{j}}+\sum _{i \in V_{l}, j \in O_{l} }{\beta _{ij}^{(k)}x_{i}x_{j}}+\sum _{i \in V_{l} \cup O_{l}}{\gamma _{i}^{(k)}x_{i}}+\delta ^{(k)} \end{aligned}$$
  - Public Key The public key *P* of Rainbow is the composition of the private keys *S*, *F*, and *T*.
    2 $$\begin{aligned} P=S \circ F \circ T: \mathbb {F}^{n}_q \rightarrow \mathbb {F}^{m}_q \end{aligned}$$
- *Signature generation* Given a message *d* to be signed and a hash function $H:\{0, 1\}^* \rightarrow \mathbb {F}^{m}_q$, the signature generation process is as follows:
  - Compute the hash value $h=H(d) \in \mathbb {F}^{m}_q$
  - Compute $$x=S^{-1}(h) \in \mathbb {F}^{m}_q$$
  - Find *y* that satisfies $F(y)=x$
  - Compute the signature $z=T^{-1}(y) \in \mathbb {F}^{n}_q$
- *Signature verification* Given a message *d* and a signature *z*, the signature verification process is as follows:
  - Compute the hash value $h=H(d) \in \mathbb {F}^{m}_q$
  - Compute the $$h'=P(z) \in \mathbb {F}^{m}_q$$
  - If $h=h'$, the signature *z* is verified

### MinRank attack

The MinRank problem asks to find coefficients $\lambda _{i} \in \mathbb {F}_{q}$ ($1 \le i \le m$), not all zero, such that the linear combination $Q=\sum _{i=1}^{m}{\lambda _{i}Q_{i}}$ has rank at most *r*, given *m* matrices $Q_{1},\cdots , Q_{m}$ with *n* rows and *n* columns and a target rank *r*. The MinRank attack is the most efficient attack for recovering the private key of Rainbow by solving the MinRank problem. The underlying idea in solving the MinRank problem is to search for a vector lying in the kernel of the desired linear combination *Q*^10^. So, the MinRank attack’s complexity is dominated by finding the kernel vector. In the case of the MinRank attack on Rainbow, it attempts to find the central map *F* of Rainbow by solving the MinRank problem. In the Rainbow scheme, a linear combination of public keys with rank $v_{2}$ corresponds to a linear combination of central maps in the first layer. By finding $o_{1}$ linear combinations, the central maps of the first layer can be reconstructed, thereby finding the private keys of Rainbow. Here, $v_{2}$ is the number of vinegar variables in the second layer , and $o_{1}$ is the number of oil variables in the first layer. Algorithm 1 shows the overall process of the MinRank attack on Rainbow^10^.

**Algorithm 1:**
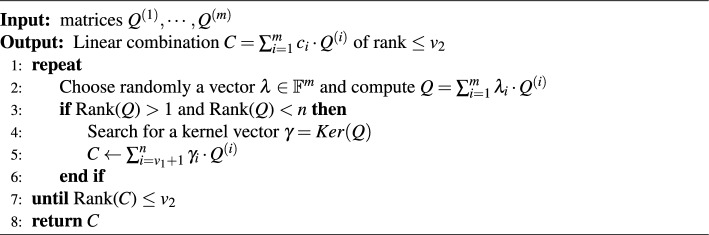
The MinRank attack.

**Figure 10:**
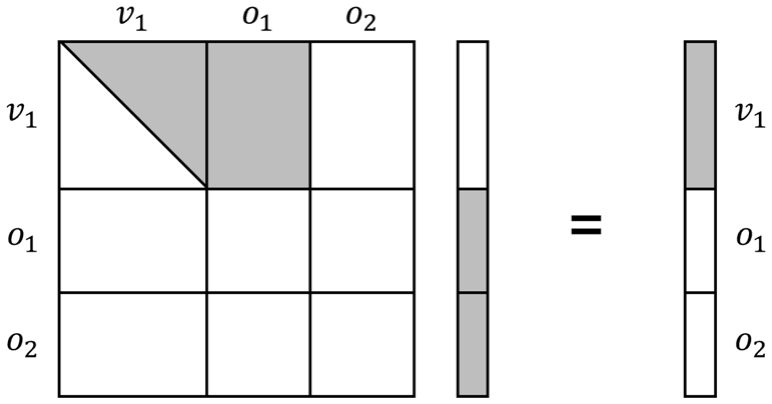
The form of kernel vectors to reduce the complexity of searching kernel.

As in line 2 of Algorithm 1, a linear combination *Q* is computed for a random vector $\lambda$ such that the rank of *P* is greater than 1 and less than *n*. The probability of finding such a vector $\lambda$ is 1/*q*, which makes the kernel vector non-trivial. The central maps of the first layer have non-zero entries at the first $v_1 \times v_1$ part, $v_1 \times o_1$ part, and $o_1 \times v_1$ part. Other parts are all zero. So the kernel vectors have only $o_1+o_2$ non-zero entries, so the central maps multiplied by the kernel vector become vectors whose last $o_1+o_2$ entries are zero, as shown in Fig. 10. Then, we must find the remaining first $v_1$ entries to be zero. The probability is close to $1/q^{v_1}$. Consequently, finding a kernel vector as shown in line 4 of Algorithm 1 takes $q^{v_1}$. Because the kernel should be non-trivial, a probability 1/*q* that the kernel is non-trivial should be considered additionally.

This process is repeated $o_1$ times to recover $o_1$ central maps. Considering all these steps, the complexity of fully recovering the first layer’s central map of Rainbow is $o_1q^{v_1+1}$. After recovering the first layer of Rainbow, an additional complexity of $m^3$ is required to recover the second layer, which is negligible compared to the complexity of recovering the first layer^10^. Finally, the complexity of the MinRank attack on Rainbow^10^ is $o_{1}\cdot q^{v_{1}+1} \cdot m^3$. A more detailed complexity analysis can be found in^10^. In this attack, it takes $q^{v_{1}}$ complexity to find a kernel of *P* (Ker(*Q*) in Algorithm 1). The complexity of finding a kernel in the parameter sets of the third round Rainbow is $2^{144}$ in parameter set I, $2^{544}$ in parameter set III, and $2^{768}$ in parameter set V.

### Grover’s algorithm

**Figure 11:**
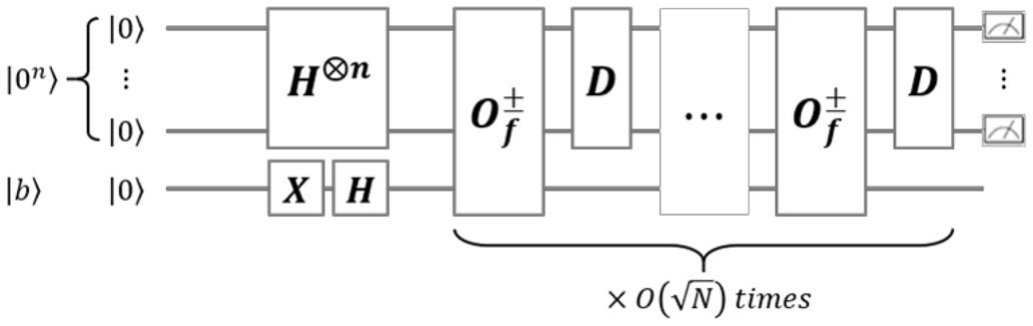
The entire circuit of the Grover’s algorithm.

An unstructured search problem is to find a solution $x^*$ in a set $\varvec{x} = {x_1, x_2,\cdots , x_N}$ such that $f(x^*) = 1$ when a boolean function $f: \varvec{x} \rightarrow \{0, 1\}$ is given. It takes *O*(*N*) complexity to solve the unstructured search problem on classical computers. Grover’s algorithm (see Algorithm 2)^22^ enables unstructured search problems to be solved with $O(\sqrt{N})$ complexity using quantum properties such as a superposition. Figure 11 shows the entire circuit of Grover’s algorithm. The circuit of Grover’s algorithm consists of a NOT gate ($\textsf{X}$), $\textsf{Hadamard}$ gates ($\textsf{H}$), oracle gates ($O_{f}^{\pm }$), and diffusion operators (*D*).

**Algorithm 2:**
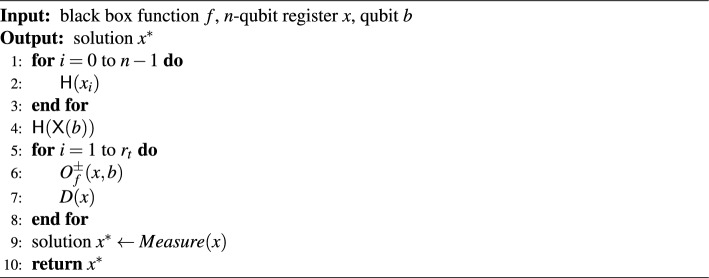
The Grover’s algorithm.

When the size of the data space is $N=2^n$, an *n*-qubit register ${|{x}\rangle }$ is prepared. After the $\textsf{Hadamard}$ gates, the state of ${|{x}\rangle }$ is converted to the superposition state ${|{x}\rangle }=2^{-n/2}\sum _{k\in \{0, 1\}^k}{|{k}\rangle }$. The oracle gate $O_{f}^{\pm }$ converts the state of ${|{x}\rangle }{|{b}\rangle }$ to ${|{x}\rangle }{|{b\oplus f(x)}\rangle }$, reversing only the amplitude of the solution $x^*$. So, the oracle gate should be designed to find a solution to the problem that we want to solve. Then, the diffusion operator $D=2{|{+^n}\rangle }{\langle {+^n}|} - I$ reverses the amplitude of $x^*$ for the mean of the amplitude of all states. As a result, the oracle gate and the diffusion operator only increase the probability that $x^*$ is measured. To greatly increase the probability that a solution will be measured, Grover’s algorithm proceeds iteratively using the oracle gate $O_{f}^{\pm }$ and Grover diffusion operator *D*. The number of iterations, $r_t$, can be selected in two ways^26^. First, if the $r_t$ is $\frac{\sqrt{N}}{8}$, the probability of measuring a solution exceeds $\frac{2}{3}$ when operating the whole Grover’s algorithm more than 110 times. Secondly, the solution will be measured with a high probability when the $r_t$ is $\frac{\pi }{4}\sqrt{N}$. In this paper, we iterate the Grover oracle and diffusion gate pair $\frac{\pi }{4}\sqrt{N}$ times to measure the solution at once.

## Quantum rectangular MinRank attack

**Figure 12:**
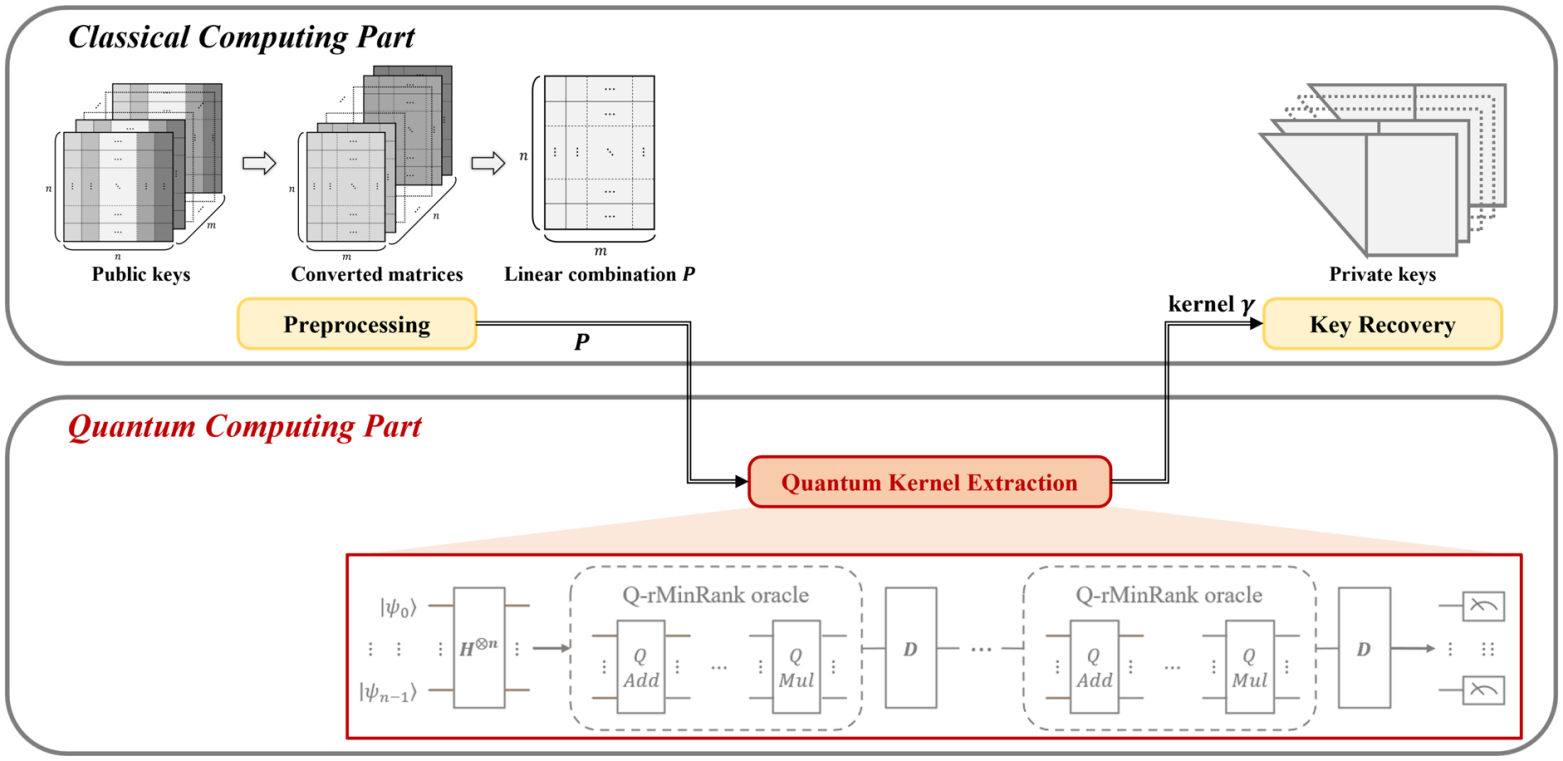
The conceptual diagram of our Q-rMinRank attack.

In this section, we propose a quantum rectangular MinRank attack, called a Q-rMinRank attack, the first quantum approach for a key recovery attack on Rainbow. The Q-rMinRank attack consists of three main steps: *preprocessing*, *quantum kernel extraction*, and *key recovery*, as shown in Fig. 12. The *preprocessing* step performs mathematical operations that convert the public keys of Rainbow into a single matrix in a classical computing environment. Then, the *quantum kernel extraction* step finds the kernel of the converted matrix using our Q-rMinRank-Grover algorithm in a quantum computing environment. Finally, the private keys are recovered by constructing a linear combination of the public keys using the kernel in the *Key Recovery* step.

The Q-rMinRank-Grover is designed to speed up the kernel search, the most complicated and time-consuming iterative operation in MinRank attacks. Since Grover’s quantum search algorithm is capable of searching in superposition states, our Q-rMinRank attack finds kernels much faster. The overall process of the Q-rMinRank attack is shown in Algorithm 3.

**Algorithm 3:**
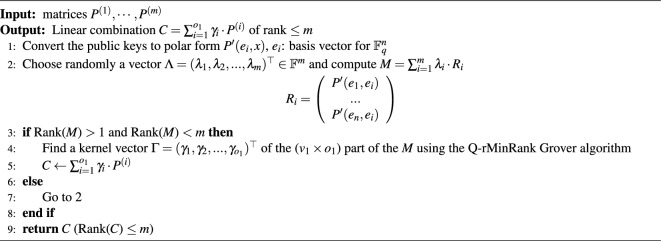
The Q-rMinRank attack.

### The *Preprocessing* Step

Rainbow has *m* public key matrices $P_1,..., P_m$ with *n* rows and *n* columns. In the *preprocessing* step, the public keys $P_1,..., P_m$ are converted to polar form. The polar form^12^ of the multivariate quadratic polynomial *p*(*x*) is defined as

3 $$\begin{aligned} p'(x, y):= p(x+y)-p(x)-p(y)+p(0). \end{aligned}$$

For a multivariate quadratic map $P(x)=p_1(x), ..., p_m(x)$, its polar form is also defined as

4 $$\begin{aligned} P'(x, y):= p'_1(x, y),..., p'_m(x,y). \end{aligned}$$

After the polar form conversion of public keys, new matrices $R_i$ are composed as follows:

5 $$\begin{aligned} R_i = \left( \begin{array}{c}P'(e_1, e_i)\\ ...\\ P'(e_n, e_i)\end{array}\right) \end{aligned}$$

where $e_i$ is a basis vector for $\mathbb {F}_{q}^{n}$, which means a vector whose *i*-th element is 1 and the rest of the elements are all zero.

**Figure 13:**
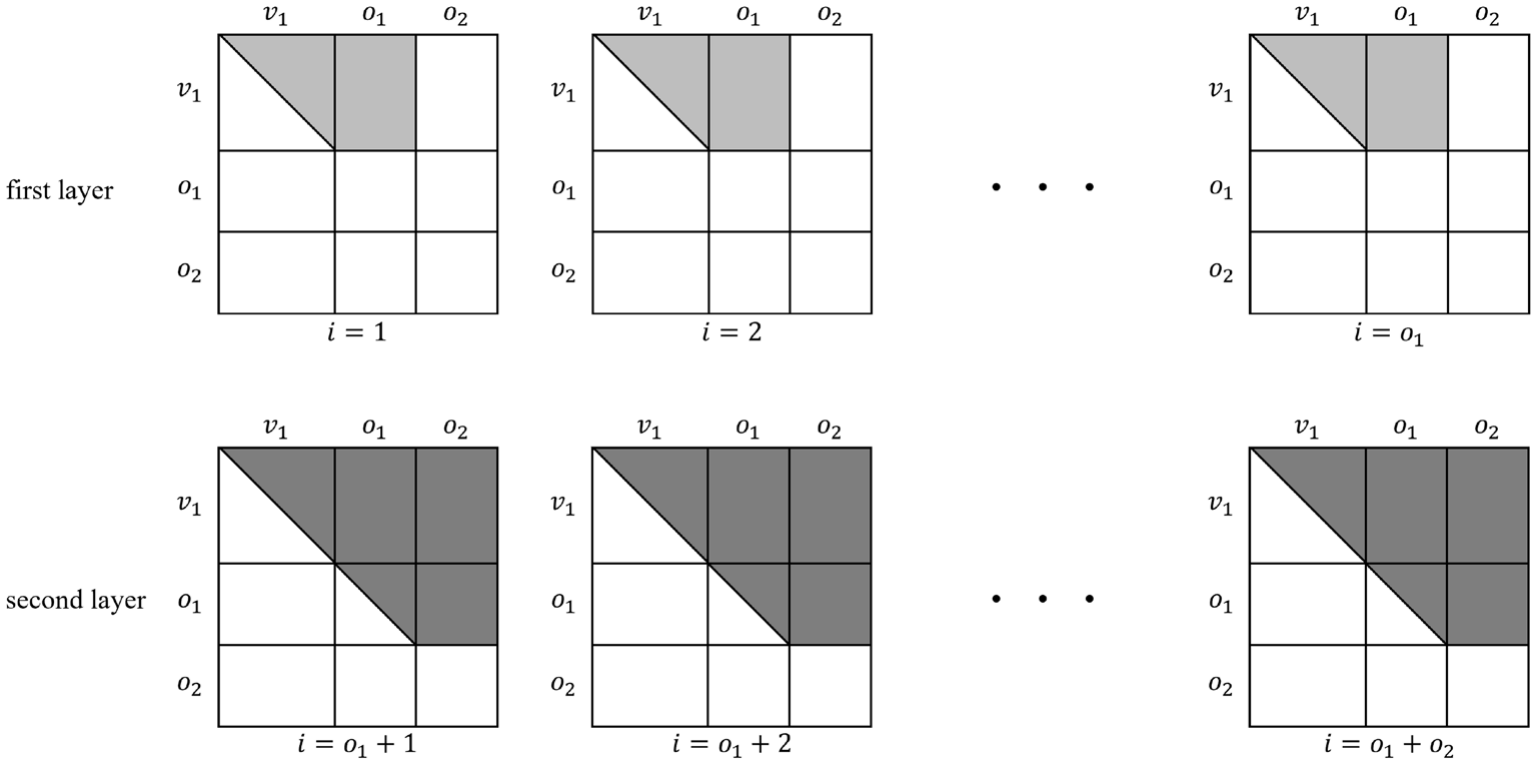
The form of public key matrices $P_1,..., P_m$.

**Figure 14:**
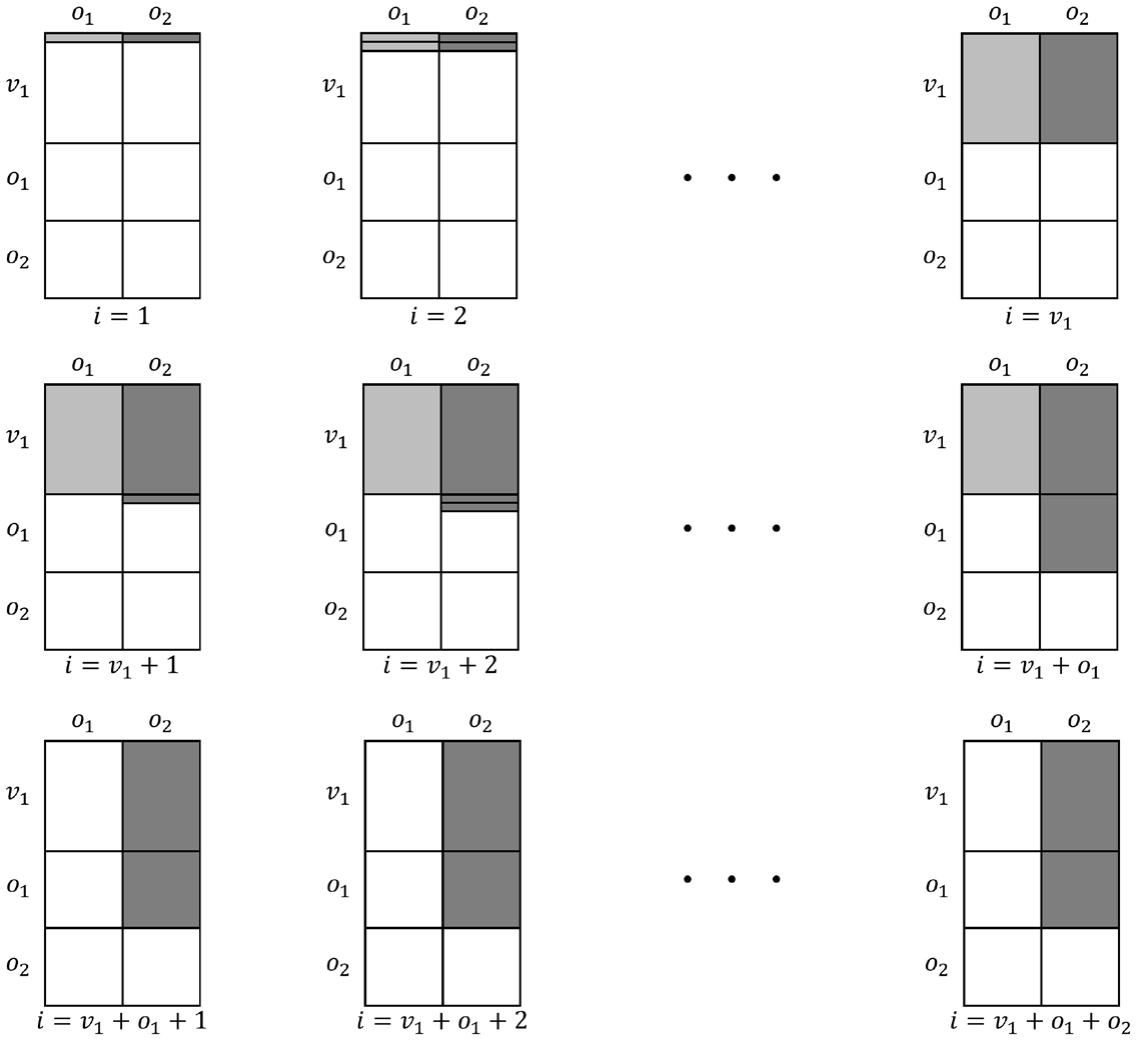
The form of matrices $R_1,..., R_n$ converted in the *preprocessing* step.

By using this polar form conversion, the *j*-th columns of the public key matrices $P_i$ with *n* rows and *n* columns ($1 \le i \le m$, see Fig. 13) are grouped into a matrix $R_j$ with *n* rows and *m* columns ($1 \le j \le n$) as shown in Fig. 14, where $n=v_1+o_1+o_2$ and $m=o_1+o_2$. Eventually, the *m* public key matrices with *n* rows and *n* columns, $P_1,...,P_m$, are converted into *n* matrices with *n* rows and *m* columns, $R_1,...,R_n$. The non-zero coefficients of the first layer exist from $R_1$ to $R_{v_1+o_1}$, as shown in Fig. 14. To recover the central map of the first layer, the linear combination *M* is computed in the *preprocessing* step by choosing a random vector $\Lambda = (\lambda _{1}, \lambda _{2}, ..., \lambda _{v_1+o_1})^\top \in \mathbb {F}^{v_1+o_1}_q$ as follows:

6 $$\begin{aligned} M=\sum _{i=1}^{v_1+o_1}{\lambda _{i}\cdot R_i} \end{aligned}$$

If the rank of *M* is zero or full, the random vector $\Lambda$ is chosen again until the rank is greater than 1 and less than *m*. The probability of this step (line 2$\scriptstyle \sim$3 in Algorithm 3) is 1/*q*. We do not consider speeding up this process with a quantum circuit, as the complexity of this part is not significant. When the rank condition is satisfied, the $v_1$-by-$o_1$ part of *M* where the coefficients of the first layer exist (i.e. the light gray-colored part of each $R_i$ in Fig. 14) is transferred to the *quantum kernel extraction* step.

7 $$\begin{aligned} M' = \left[ \begin{array}{ccc}M_{0, 0} &{} \hdots &{} M_{0, (o_1-1)} \\ \vdots &{} \ddots &{} \vdots \\ M_{(v_1-1), 0} &{} \hdots &{} M_{(v_1-1), (o_1-1)} \\ \end{array}\right] \end{aligned}$$

### The *Quantum Kernel extraction* Step

To find a kernel of the matrix $M'$ with $v_1$ rows and $o_1$ columns, a Q-rMinRank-Grover algorithm (see Algorithm 4) is performed in the *Quantum Kernel Extraction* step. The Q-rMinRank-Grover algorithm requires quantum registers ${|{x_0}\rangle }$ $\scriptstyle \sim$ ${|{x_{o_1-1}}\rangle }$, ${|{e_0}\rangle }$ $\scriptstyle \sim$ ${|{e_{v_1-1}}\rangle }$, a qubit ${|{b}\rangle }$, and some ancilla qubits ${|{anc}\rangle }$. The quantum registers ${|{x_0}\rangle }$ $\scriptstyle \sim$ ${|{x_{o_1-1}}\rangle }$ and ${|{e_0}\rangle }$ $\scriptstyle \sim$ ${|{e_{v_1-1}}\rangle }$ are $\log _{2}{q}$-qubit registers, where $\log _{2}{q}$ is the number of qubits required to express a number on $\mathbb {F}_q$. The required quantum registers and their roles are shown in Table 3.

**Algorithm 4:**
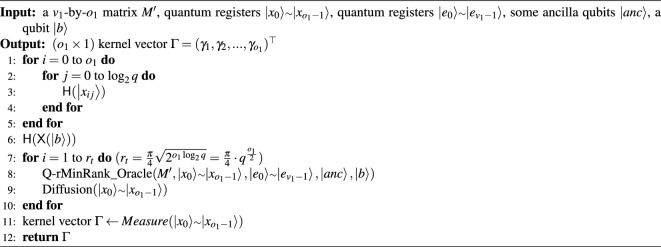
The Q-rMinRank-Grover algorithm.

**Table 3:** The quantum registers used in our Q-rMinRank-Grover algorithm.

Quantum registers	Number of qubits	Role of quantum registers
${|{x_0}\rangle }$ $\scriptstyle \sim {|{x_{o_1-1}}\rangle }$	$\log _{2}{q}$	At the end of the Q-rMinRank-Grover algorithm, the kernel is measured at quantum registers ${|{x_0}\rangle }$ $\scriptstyle \sim$ ${|{x_{o_1-1}}\rangle }$.
${|{e_0}\rangle }\scriptstyle \sim {|{e_{v_1-1}}\rangle }$	$\log _{2}{q}$	The sum of multiplications between each row of the input matrix and quantum registers ${|{x_0}\rangle }$ $\scriptstyle \sim$ ${|{x_{o_1-1}}\rangle }$ is stored in quantum registers ${|{e_0}\rangle }$ $\scriptstyle \sim$ ${|{e_{v_1-1}}\rangle }$.
${|{b}\rangle }$	1	The qubit ${|{b}\rangle }$ is reversed when the state of quantum registers ${|{e_0}\rangle }$ $\scriptstyle \sim$ ${|{e_{v_1-1}}\rangle }$ are all 0.

Firstly, the Hadamard gates in the Q-rMinRank-Grover algorithm bring all the qubits in quantum registers ${|{x_0}\rangle }$ $\scriptstyle \sim$ ${|{x_{o_1-1}}\rangle }$ and a qubit ${|{b}\rangle }$ into superposition states. Then, to increase the probability of measuring the kernel, the pair of Q-rMinRank_Oracle($\cdot$) and diffusion circuit *Diffusion*($\cdot$) operates iteratively $r_t$ times. The Q-rMinRank_Oracle($\cdot$) function checks which state of superpositioned quantum registers ${|{x_0}\rangle }$ $\scriptstyle \sim$ ${|{x_{o_1-1}}\rangle }$ is the kernel for the matrix $M'$. The quantum registers ${|{e_0}\rangle }$ $\scriptstyle \sim$ ${|{e_{v_1-1}}\rangle }$ contain the multiplication of matrix $M'$ and the kernel vector. If all the registers from ${|{e_0}\rangle }$ to ${|{e_{v_1-1}}\rangle }$ are zero, Q-rMinRank_Oracle($\cdot$) reverses the qubit ${|{b}\rangle }$. The quantum circuit for the Q-rMinRank_Oracle($\cdot$) function can be designed differently for purposes such as depth optimization and width optimization. The details of quantum circuit design for the Q-rMinRank_Oracle($\cdot$) function are covered in the “Oracle circuit designs” section.

**Algorithm 5:**
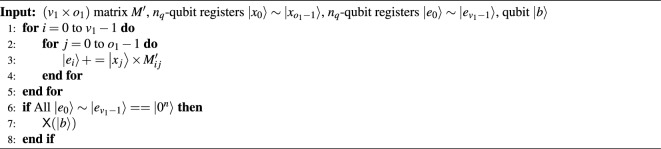
The Q-rMinRank_Oracle($\cdot$) function.

According to the available quantum resources, the number of iterations $r_t$ can be adjusted^26^. The $r_t$ may be selected as $\frac{1}{8}\sqrt{2^{o_1\log _{2}{q}}}$ when there are few available quantum resources. In that case, we measure a kernel with a probability of more than $\frac{2}{3}$ when we run the entire Q-rMinRank Grover circuit about 110 times. If quantum resources are sufficient, the $r_t$ may be selected as $\frac{\pi }{4}\sqrt{2^{o_1\log _{2}{q}}}$. In this case, we find the kernel with a probability of more than 90% by running the Q-rMinRank Grover circuit once.

### The *Key recovery* Step

After the kernel vector, $\Gamma$ is obtained in the *quantum kernel extraction* step, the *key recovery* step recovers the Rainbow central map of the first layer by computing the linear combination *C* of $o_1$ public keys with $\Gamma$ again as follows:

8 $$\begin{aligned} \displaystyle C\leftarrow \sum _{i=1}^{o_1} \gamma _{i}\cdot P^{(i)} \end{aligned}$$

Generally, the rank of the linear combination *C* is less than *m* with a high probability (over 90% on our quantum simulation). If the rank of *C* is greater than *m*, the *preprocessing* step and the *quantum kernel extraction* step are repeated until the rank of *C* is less than *m*. By finding ($v_1+o_1$) linear combinations *C* that have a rank lower than *m* in the *quantum kernel extraction* step, all $o_1$ central maps of the first layer are recovered. Considering that the public key *P* is composed of $S \circ F \circ T : \mathbb {F}^{n}_q \rightarrow \mathbb {F}^{m}_q$, *T* is uncovered from *P* using the central map recovered in the *key recovery* step. After that, the central maps of the second layer are recovered by uncovering *T* and *F* from the public key^10^.

## Oracle circuit designs

In this section, we present the Q-rMinRank_Oracle circuit, the main quantum circuit, to recover the private key of Rainbow by quickly finding the kernel. We consider two important metrics, circuit depth and circuit width, to design quantum circuits in available quantum computing environments. For quantum security analysis of post-quantum cryptography, NIST has defined a method for measuring the quantum complexity (QC) by multiplying the quantum circuit depth by the number of circuit gates (G-cost)^16^. Since the number of qubits available is rapidly increasing with the development of quantum computers, NIST is considering only the depth and G-cost that affect the running time of quantum circuits rather than the width (number of qubits, #QB). However, the width of circuits is directly related to the feasibility of implementing quantum circuits and running them on quantum computers. So, width is still one of the essential metrics for estimating the performance of quantum circuits.

Therefore, we present two quantum oracle circuits, $\mathcal {O}_1$ and $\mathcal {O}_2$, considering the depth-width trade-off. $\mathcal {O}_1$ is a depth-optimized version of the Q-rMinRank_Oracle, and $\mathcal {O}_2$ is a width-optimized version of the Q-rMinRank_Oracle. For the design of $\mathcal {O}_1$, we use a method to parallelize the quantum arithmetic gates ($\textsf{MULT}$ and $\textsf{ADD}$). Parallelization allowed us to reduce the depth of $\mathcal {O}_1$. We implement gates with minimal ancilla qubits without parallelization for the design of $\mathcal {O}_2$. So we could reduce the width of $\mathcal {O}_2$.

### The depth-optimized Q-rMinRank_Oracle $\mathcal {O}_1$

**Figure 15:**
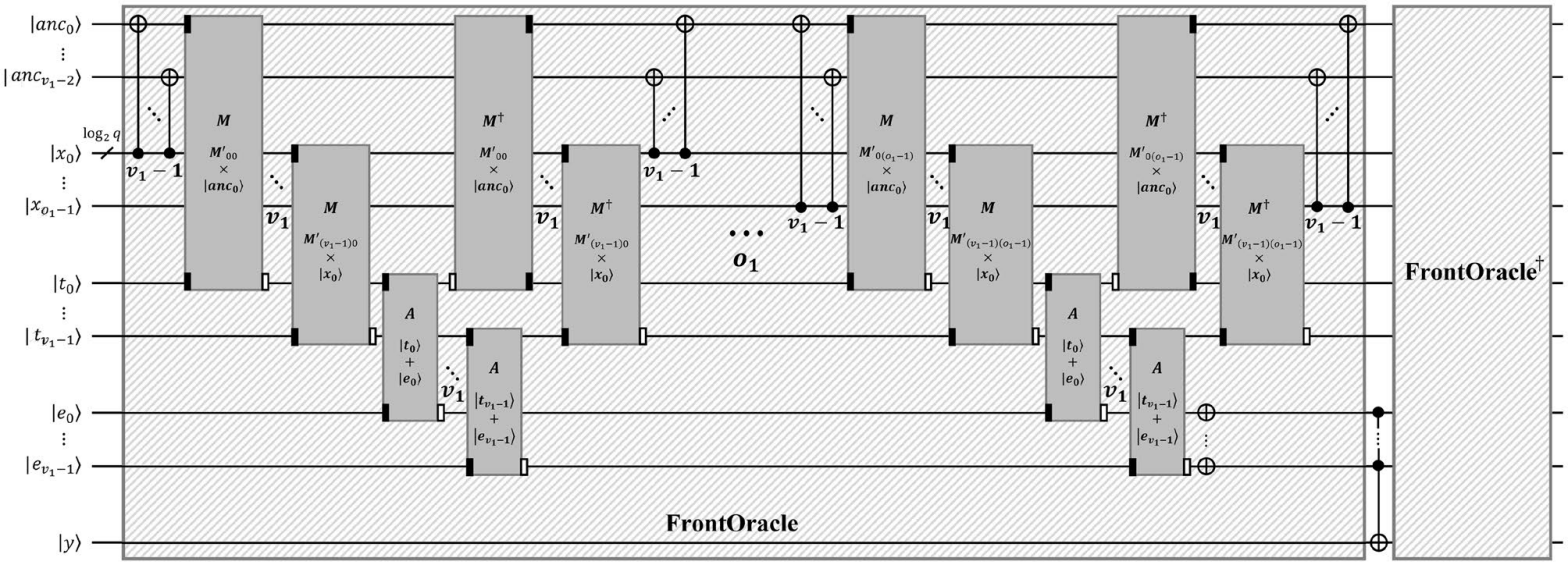
The depth-optimized Q-rMinRank_Oracle $\mathcal {O}_1$.

The Q-rMinRank_Oracle $\mathcal {O}_1$ parallelizes the $\textsf{MULT}$ and $\textsf{ADD}$ gates, as shown in Algorithm 6. It is necessary to check whether the linear combination of the values in the *i*-th column and quantum registers ${|{x_i}\rangle }$ is zero for each row of the matrix to find a kernel of the input matrix $M'$. Firstly, the $\textsf{CNOT}$ gates copy the state of quantum register ${|{x_0}\rangle }$ to $v_1-1$ ancilla quantum registers ${|{anc_j}\rangle }$ $(0 \le j < v_1-1)$ so that the $v_1$ states of ${|{x_0}\rangle }$ are prepared. Assigning more ancilla qubits in this way enables the parallelization of $\textsf{MULT}$ gates. Then, $v_1$ $\textsf{MULT}$ gates operate in parallel to multiply the qubits by the elements of the matrix, $M'_{0,0}$ $\scriptstyle \sim$ $M'_{(v_1-1),0}$, and store the multiplication results in ${|{t_0}\rangle }$ $\scriptstyle \sim$ ${|{t_{v_1-1}}\rangle }$. The states of ${|{t_0}\rangle }$ $\scriptstyle \sim$ ${|{t_{v_1-1}}\rangle }$ are added to ${|{e_0}\rangle }$ $\scriptstyle \sim$ ${|{e_{v_1-1}}\rangle }$ using the $\textsf{ADD}$ gates also in parallel. After that, $v_1$ $\textsf{MULT}^{\dagger }$ and $\textsf{CNOT}$ gates operate to initialize the ${|{t_0}\rangle }$ $\scriptstyle \sim$ ${|{t_{v_1-1}}\rangle }$ and ${|{anc_0}\rangle }$ $\scriptstyle \sim$ ${|{anc_{v_1-2}}\rangle }$ in parallel, respectively, as shown in Fig. 15. The total number of ancilla qubits required for parallelization is $v_1o_1\log _{2}{q}$. The above process ($\textsf{MULT}$, $\textsf{ADD}$, and $\textsf{MULT}^{\dagger }$ pairs) is then iterated $o_1$ times to store all states of the quantum registers ${|{e_i}\rangle }$.

**Algorithm 6:**
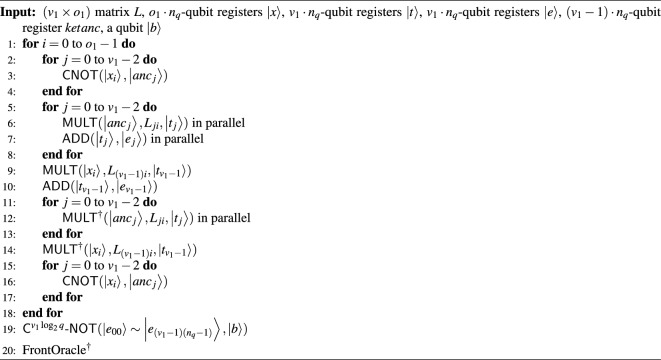
The depth-optimized Q-rMinRank_Oracle $\mathcal {O}_1$.

In order to implement oracle $\mathcal {O}_1$, we use the quantum multiplier^25^(see Fig. 8), which has a smaller depth, as shown in Table 2. Moreover, we implement the $\textsf{C}^{v_1\log _{2}{q}}$-$\textsf{NOT}$ gate as $\textsf{C}^{v_1\log _{2}{q}}$-$\textsf{NOT}_\textsf{D}$ in Fig. 5a to shorten the depth of oracle $\mathcal {O}_1$. Since the implementation of $\textsf{C}^{v_1\log _{2}{q}}$-$\textsf{NOT}_\textsf{D}$ requires $v_1\log _{2}{q}$ ancilla qubits, as shown in Table 1, the ancilla qubits for parallelization can be reused.

#### The width-optimized Q-rMinRank_Oracle $\mathcal {O}_2$

**Figure 16:**
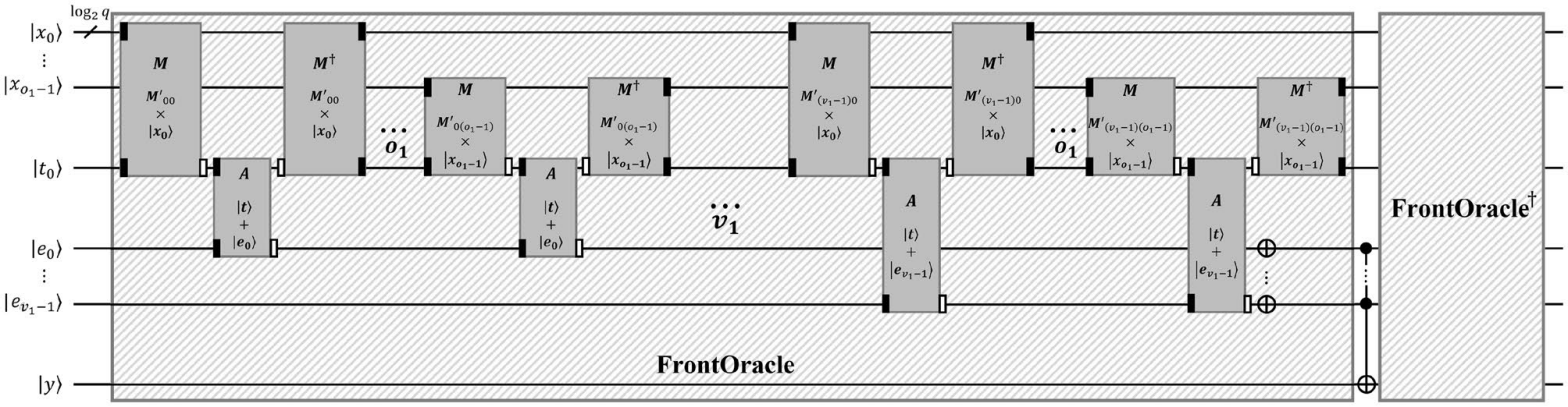
The width-optimized Q-rMinRank_Oracle $\mathcal {O}_2$.

The Q-rMinRank_Oracle $\mathcal {O}_2$ has no additional ancilla qubits needed for parallelization. Thus, $\mathcal {O}_2$ can reduce the number of qubits. Algorithm 7 shows the process of oracle $\mathcal {O}_2$, in which $\textsf{MULT}$ and $\textsf{ADD}$ gates operate linearly. We utilize the quantum multiplier^24^ as the $\textsf{MULT}$ gate (see Fig. 7), which uses smaller ancilla qubits as shown in Table 2. After the $\textsf{MULT}$ gate multiplies $M'_{0,0}$ by the quantum register ${|{x_0}\rangle }$ and stores the multiplication results in the quantum register ${|{t}\rangle }$, the $\textsf{ADD}$ gate operates to add the state of ${|{t}\rangle }$ to ${|{e_0}\rangle }$. The $\mathsf {{MULT}}^{\dagger }$ gate operates to initialize the ${|{t}\rangle }$. The $\mathcal {O}_2$ iterates $\textsf{MULT}$, $\textsf{ADD}$, and $\textsf{MULT}^{\dagger }$ pairs (called $\textsf{MAM}^{\dagger }$ pairs) $o_1$ times to construct the ${|{e_i}\rangle }$ as shown in Fig. 16. As $\mathcal {O}_2$ has to construct $v_1$ quantum registers ${|{e_i}\rangle }$ ($0\le i <v_1$), the $\mathcal {O}_2$ totally iterates $\textsf{MAM}^{{\dagger }}$ pairs ($v_1\times o_1$) times without parallel operations. During this process, $\mathcal {O}_2$ does not use ancilla qubits ${|{anc}\rangle }$, which temporarily stores the multiplication result by $\textsf{MULT}$ gate.

**Algorithm 7:**
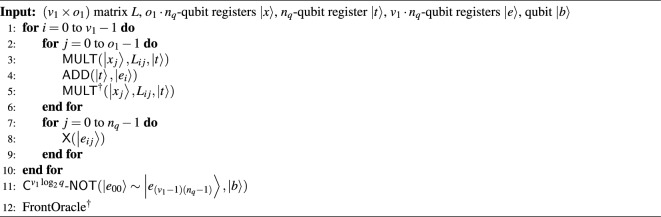
The width-optimized Q-rMinRank_Oracle $\mathcal {O}_2$.

since $\mathcal {O}_2$ does not perform parallelization between quantum arithmetic gates, it has quite a long depth. The depth of $\mathcal {O}_2$ is computed by multiplying the depth of the $\textsf{MAM}^{\dagger }$ pair by ($v_1 \times o_1$). We further reduce the number of qubits by implementing the $\textsf{C}^{v_1\log _{2}{q}}$-$\textsf{NOT}$ gate as $\textsf{C}^{v_1\log _{2}{q}}$-$\textsf{NOT}_\textsf{W}$ in Fig. 5b. As shown in Table 1, $\textsf{C}^{v_1\log _{2}{q}}$-$\textsf{NOT}_\textsf{W}$ requires only one ancilla qubit to implement the $\textsf{C}^{v_1\log _{2}{q}}$-$\textsf{NOT}$ gate. By recycling an ancilla qubit in the quantum register ${|{t_0}\rangle }$, we construct $\mathcal {O}_2$ without using additional ancilla qubits.

## Complexity analysis

In this section, we analyze the complexity of the Q-rMinRank attack for each parameter set of third-round Rainbow and evaluate the security level of Rainbow. Then, we analyze the quantum resources required for the Q-rMinRank attack.

### Security analysis

The complexity of our Q-rMinRank attack depends on the size of the kernel vector space. We reduced the complexity of finding a kernel by converting the *m* $(n \times n)$ public keys of Rainbow to the *n* $(n \times m)$ matrices using polar form conversion in the preprocessing step. The Rainbow signature scheme has three security levels, with each parameter set as shown in Table 4.

**Table 4:** Description of the NIST security categories and relation with the Rainbow security levels.

Rainbow parameter sets					NIST secuirty level categories	
Parameter sets	*q*	$v_1$	$o_1$	$o_2$	Security levels	Descriptions
I	$2^4$	36	32	32	$2^{128}$	Hard to break AES128
II	$2^8$	68	32	48	$2^{192}$	Hard to break AES192
V	$2^8$	96	36	64	$2^{256}$	Hard to break AES256

*q*: the number of elements in the finite field

$v_1$: the number of vinegar variables of the first layer

$o_1$: the number of oil variables of the first layer

$o_2$: the number of oil variables of the second layer.

#### Proposition 1

In our Q-rMinRank attack, the complexity of recovering the first layer of Rainbow is $\frac{\pi }{4}q(v_1+o_1)\sqrt{2^{o_1n_q}}$.

#### Proof

When the matrices $P_1,..., P_m$ are given as Fig. 13 and the matrices are converted to $R_i$ according to equations (3)$\scriptstyle \sim$(5), the size of the *n* converted matrices $R_i$ ($0 \le i <n$) is ($n \times m$) as shown in Fig. 14. The information in the first layer exists from $R_1$ to $R_{(v_1+o_1)}$. To recover the central map of the first layer, we only use the ($v_1 \times o_1$) part of $R_i$, which has information about the first layer. In our Q-rMinRank attack, we only need to find a kernel vector $\Gamma \in \mathbb {F}_q^{o_1}$ for the linear combination $M'$ such that $\Gamma \in Ker{M'}$, when the $M'$ is a linear combination of parts of ($v_1 \times o_1$) from $R_1$ to $R_{(v_1+o_1)}$. Since the size of the kernel is $o_1$, the number of qubits needed to construct the Grover quantum circuit is $o_1\times n_q$. After computing the linear combination *M* that satisfies line 3 of Algorithm 3 (with a complexity of *q*), our Q-rMinRank Grover algorithm finds the kernel with a complexity of $\frac{\pi }{4}\sqrt{2^{o_1n_q}}$. We have to find $v_1+o_1$ kernels, so the total complexity of recovering the first layer of Rainbow is

9 $$\begin{aligned} \frac{\pi }{4}q(v_1+o_1)\sqrt{2^{o_1n_q}}. \end{aligned}$$

$$\square$$

#### Proposition 2

In our Q-rMinRank attack, the complexity of recovering the second layer of Rainbow is negligible.

#### Proof

A canonical form $g(z_1,..., z_r)$ is defined by $z_1z_2+\cdot \cdot \cdot + z_{r-2}z_{r-1}+z_r^2$ when *r* is odd or $z_1z_2+\cdot \cdot \cdot + z_{r-1}z_{r}+b(z_{r-1}^2+az_r^2)$ when *r* is even. Then, given a quadratic form $f \in \mathbb {F}_q[x_1, ..., x_n]$ of rank *r*, there exists a matrix *G* of rank *r* mapping $(x_1,..., x_n)$ to $(z_1,..., z_r)$ such that

10 $$\begin{aligned} f(x_1,..., x_n) = g \cdot G(x_1,..., x_n). \end{aligned}$$

We can find the matrix *G* using a deterministic algorithm with a complexity lower than $n^3$. As a result, we uncover *T* with a complexity of $n^3$. Since we know the central maps of the first layer recovered from the Q-rMinRank attack, public keys, and another private key *T*, we can recover the last private key *S* as follows:

11 $$\begin{aligned} S = P(TFT^T)^{-1} \end{aligned}$$

Then, we recover the central map of the second layer simply by matrix operations ($F = T^{-1}S^{-1}P(T^T)^{-1}$). As both the matrix operations and the matrix inversion require a complexity of $n^3$, the additional cost of recovering the central map of the second layer is dominated by $O(n^3)$ and is negligible^10^. $\square$

From Propositions 1 and 2, we see that the complexity of the Q-rMinRank attack depends on the cost of recovering the central map of the first layer. Since the $o_1$ central maps of the first layer are recovered by iterating the *quantum kernel extraction* step $v_1 + o_1$ times, our attack complexity is $\frac{\pi }{4}(v_1+o_1)\sqrt{2^{o_1n_q}}$. Table 5 compares the complexity of our Q-rMinRank attack with the Rainbow team’s quantum approach for the MinRank attack applying the Grover algorithm^10^.

**Table 5:** A complexity comparison of the MinRank attack^10^, the Rectangular MinRank attack^12^, the improved Rectangular MinRank attacks^13^, and our Q-rMinRank attack.

Rainbow parameter sets					Complexities of quantum attack	
Parameter sets	*q*	$v_1$	$o_1$	$o_2$	Grover on^10^	our Q-rMinRank attack
I	$2^4$	36	32	32	$2^{97}$	$2^{71}$
II	$2^8$	68	32	48	$2^{303}$	$2^{140}$
V	$2^8$	96	36	64	$2^{416}$	$2^{156}$

*q*: the number of elements in the finite field

$v_1$: the number of vinegar variables of the first layer

$o_1$: the number of oil variables of the first layer

$o_2$: the number of oil variables of the second layer

### Quantum resource analysis

For quantum resource analysis, we estimated the number of qubits (#QB), the number of quantum gates (G-cost), and the quantum depth (*D*) required to perform our Q-rMinRank-Grover algorithm. Our Q-rMinRank-Grover algorithm consists of iterations of Q-rMinRank_Oracle and the diffusion operator.

The diffusion operator’s circuit is fixed according to the number of input qubits. The G-cost used in the diffusion operator is proportional to the number of iterations $r_t$, so it is not negligible. Therefore, we estimated the quantum resources of the oracle circuit and the diffusion operator for more accurate analysis. Table 6 shows the quantum resources for our Q-rMinRank_Oracle and Diffusion pair. The #QB required for the $\mathcal {O}_2$ is one-fourth of $\mathcal {O}_1$ for parameter set I, one-seventh of $\mathcal {O}_1$ for parameter set III, and one-eighth of $\mathcal {O}_1$ for parameter set V. On the other hand, $\mathcal {O}_1$ has a depth of about $2^6$ to $2^9$ smaller than $\mathcal {O}_2$. $\mathcal {O}_1$ and $\mathcal {O}_2$ have a trade-off between the number of qubits and depth. For the cost comparison of $\mathcal {O}_1$ and $\mathcal {O}_2$ implementations, we also present the qubit-cycle costs of each oracle. The total cost in logical qubit-cycles for the serial overhead comparison is $C=DW$, while the cost in qubit-cycles for the parallel overhead comparison is $C=D^2W$^27^. The $\mathcal {O}_1$ implementation uses more qubits but has lower overhead due to the depth optimization.

**Table 6:** The quantum resources for our Q-rMinRank_Oracle and Diffusion pair.

Algorithm	Parameter sets	Oracle	#QB	G-cost	*D*	*DW*	$D^2W$
Rainbow	I	$\mathcal {O}_1$	1097	$1.74 \times 2^{18}$	$1.09 \times 2^{11}$	$2^{21.2}$	$2^{32.3}$
		$\mathcal {O}_2$	289	$1.42 \times 2^{19}$	$1.14 \times 2^{17}$	$2^{25.4}$	$2^{42.5}$
	III	$\mathcal {O}_1$	5757	$1.37 \times 2^{21}$	$1.47 \times 2^{11}$	$2^{24.0}$	$2^{35.6}$
		$\mathcal {O}_2$	833	$1.34 \times 2^{22}$	$1.60 \times 2^{19}$	$2^{29.4}$	$2^{49.1}$
	V	$\mathcal {O}_1$	8057	$1.09 \times 2^{22}$	$1.65 \times 2^{11}$	$2^{24.7}$	$2^{36.4}$
		$\mathcal {O}_2$	1089	$1.06 \times 2^{23}$	$1.27 \times 2^{20}$	$2^{30.4}$	$2^{50.8}$

G-cost: the number of quantum gates used in an oracle and diffusion pair

*D*: the depth for an oracle and diffusion pair

*DW*: the depth times width for an oracle and diffusion pair (serial overhead)

$D^2W$: the square of depth times width for an oracle and diffusion pair (parallel overhead).

Then, we evaluated the efficiency of our circuits in terms of the quantum complexity proposed by NIST as a metric that should be considered in analyzing the security against quantum attacks on PQCs. Considering future quantum computers’ performance, NIST proposes measuring the complexity of quantum attacks in terms of a restricted circuit depth called the MAXDEPTH. Table 7 shows the plausible values for the MAXDEPTH range provided by NIST.

**Table 7:** The plausible MAXDEPTH range^16^.

MAXDEPTH	Description
$2^{40}$	The approximate number of gates that presently envisioned quantum computing architectures are expected to serially perform in a year
$2^{64}$	The approximate number of gates that current classical computing architectures can perform serially in a decade
$2^{96}$	The approximate number of gates that atomic scale qubits with speed of light propagation times could perform in a millennium

Based on our quantum resource estimation and MAXDEPTH values, we analyzed the quantum complexity of our Q-rMinRank-Grover algorithm. Table 8 shows the G-cost by MAXDEPTH and the quantum complexity of our Q-rMinRank-Grover. The quantum complexity is approximated as follows:

$${Complexity}_{quantum}$$ = G-cost $$\times$$ *D*

**Table 8:** The G-cost by MAXDEPTH and the quantum complexity of Q-rMinRank-Grover.

Algorithm	Parameter sets	Oracle	MD	G-cost	*D*	$Complexity_{quantum}$
Rainbow	I	$\mathcal {O}_1$	$2^{40}$	$1.90 \times 2^{53}$	$1.00 \times 2^{40}$	$1.90 \times 2^{93}$ / MAXDEPTH
			$2^{64}$	$1.90 \times 2^{29}$	$1.00 \times 2^{64}$	
			$2^{96}$	$1.74 \times 2^{18}$	$1.09 \times 2^{75}$	
		$\mathcal {O}_2$	$2^{40}$	$1.63 \times 2^{60}$	$1.00 \times 2^{40}$	$1.63 \times 2^{100}$ / MAXDEPTH
			$2^{64}$	$1.63 \times 2^{36}$	$1.00 \times 2^{64}$	
			$2^{96}$	$1.63 \times 2^{4}$	$1.00 \times 2^{96}$	
	III	$\mathcal {O}_1$	$2^{40}$	$1.01 \times 2^{118}$	$1.00 \times 2^{40}$	$1.01 \times 2^{158}$ / MAXDEPTH
			$2^{64}$	$1.01 \times 2^{94}$	$1.00 \times 2^{64}$	
			$2^{96}$	$1.01 \times 2^{62}$	$1.00 \times 2^{96}$	
		$\mathcal {O}_2$	$2^{40}$	$1.07 \times 2^{127}$	$1.00 \times 2^{40}$	$1.07 \times 2^{167}$ / MAXDEPTH
			$2^{64}$	$1.07 \times 2^{103}$	$1.00 \times 2^{64}$	
			$2^{96}$	$1.07 \times 2^{71}$	$1.00 \times 2^{96}$	
	V	$\mathcal {O}_1$	$2^{40}$	$1.80 \times 2^{134}$	$1.00 \times 2^{40}$	$1.80 \times 2^{174}$ / MAXDEPTH
			$2^{64}$	$1.80 \times 2^{110}$	$1.00 \times 2^{64}$	
			$2^{96}$	$1.80 \times 2^{78}$	$1.00 \times 2^{96}$	
		$\mathcal {O}_2$	$2^{40}$	$1.35 \times 2^{144}$	$1.00 \times 2^{40}$	$1.35 \times 2^{184}$ / MAXDEPTH
			$2^{64}$	$1.35 \times 2^{120}$	$1.00 \times 2^{64}$	
			$2^{96}$	$1.35 \times 2^{88}$	$1.00 \times 2^{96}$	

## Quantum simulation result

To show the feasibility of our Q-rMinRank attack, we implemented and simulated toy example circuits for our Q-rMinRank-Grover algorithms, where oracle consists of $\mathcal {O}_1$ and $\mathcal {O}_2$, respectively. The input matrix for the toy example is given on $\mathbb {F}_8$ as follows:

12 $$\begin{aligned} \begin{bmatrix} 5 &{} 1 \\ 7 &{} 5 \\ 4 &{} 3 \end{bmatrix} \end{aligned}$$

We constructed a circuit of the Q-rMinRank-Grover algorithm that finds the kernel of the matrix (Equation 12) and simulated our circuit using quantum simulators Qiskit^20^ and ProjectQ^21^.

**Figure 17:**
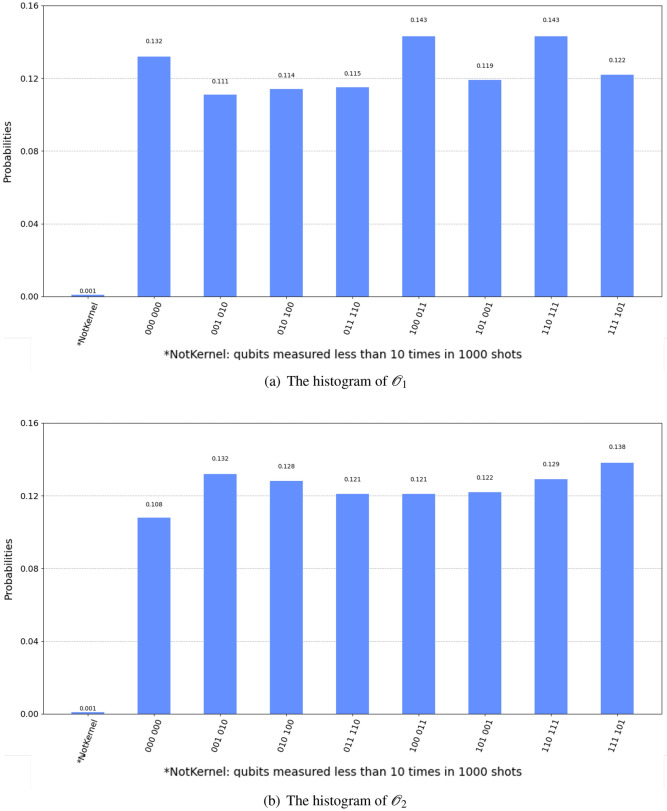
The histogram obtained by running our Q-rMinRank-Grover with oracles $\mathcal {O}_1$ and $\mathcal {O}_2$ in Qiskit Aer simulator.

**Figure 18:**
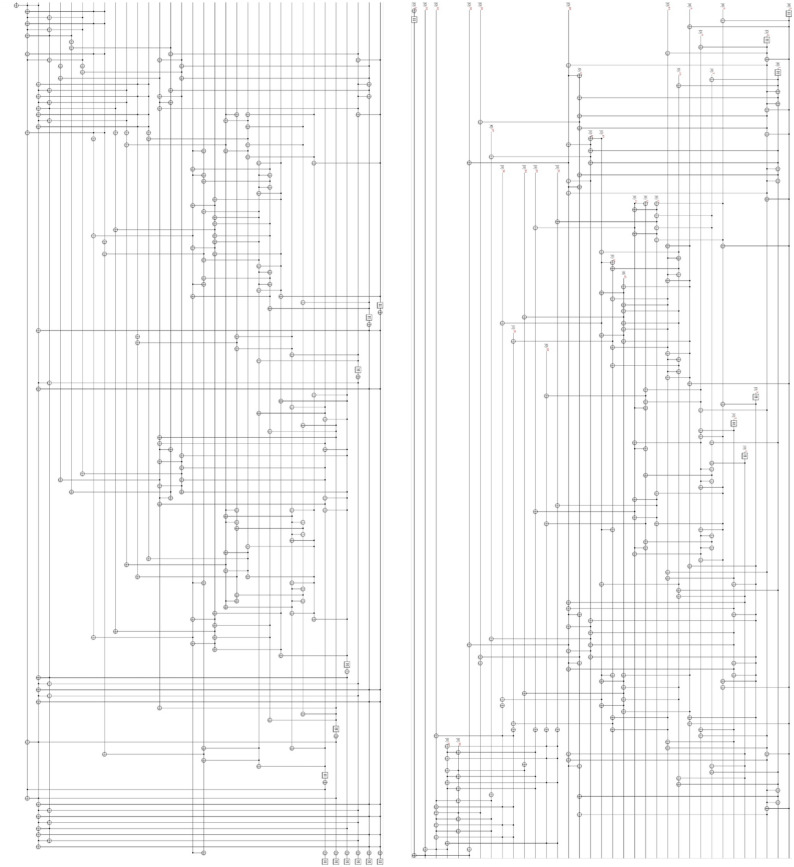
Q-rMinRank-Grover algorithm quantum circuit for toy example of “Quantum simulation result” section when the Q-rMinRank_Oracle $\mathcal {O}_1$ and diffusion circuit pairs repeat 1.

**Figure 19:**
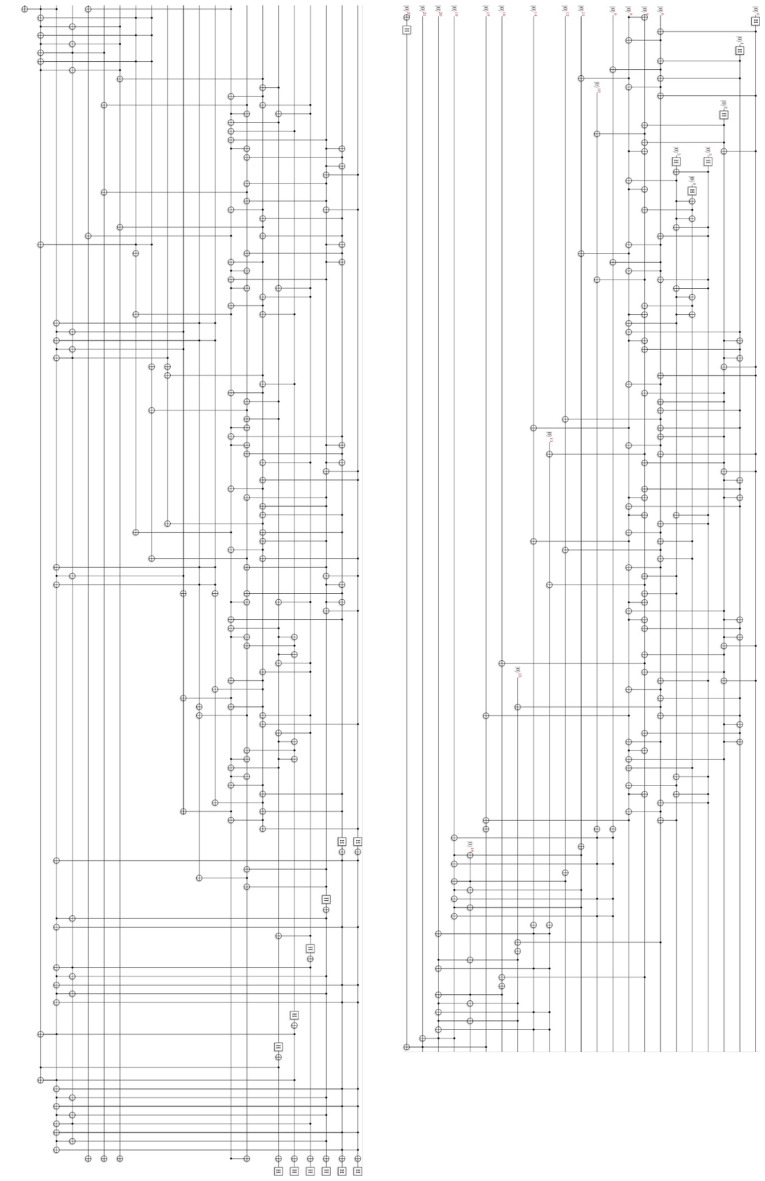
Q-rMinRank-Grover algorithm quantum circuit for toy example of “Quantum simulation result” section when the Q-rMinRank_Oracle $\mathcal {O}_2$ and diffusion circuit pairs repeat 1.

ProjectQ, an open-source software effort for quantum computing, provides a function for drawing quantum circuits. Figures 18 and 19 show our Q-rMinRank oracle quantum circuits of the Q-rMinRank attack drawn using ProjectQ. Qiskit, another open-source framework for quantum computing, has the advantage of fast execution speed and supports multi-shot simulation that shows the frequency of measurement when executed several times. We measured the probability that our Q-rMinRank-Grover algorithms find the kernel utilizing the multi-shot function of Qiskit. Figures 17a and b show the outputs of implementing our Q-rMinRank-Grover algorithm circuits for the toy example in the Qiskit Aer simulator for 1000 shots.

Qubit values measured less than 10 times are not kernel values, and the sum of their probabilities is indicated in the bar named ’NotKernel’. Our simulation results show that the ’NotKernel’ is measured only once out of 1000 times. The other bars represent each of the probabilities when the kernels are accurately measured. Since we need to get a non-zero kernel, we must also exclude the results measured as ’000 000’. The Q-rMinRank-Grover algorithms, consisting of oracles $\mathcal {O}_1$ and $\mathcal {O}_2$, respectively, find the kernel with accuracies of 86.7% and 89.1% probability, respectively.

## Conclusion

In this paper, we first proposed a quantum rectangular MinRank (Q-rMinRank) attack that applied quantum algorithms to key recovery attacks against Rainbow, especially MinRank attacks. We designed oracle $\mathcal {O}_1$ optimized for the quantum depth and oracle $\mathcal {O}_2$ optimized for the quantum width (the number of qubits), respectively, considering quantum computing environments. According to our quantum resource estimation, the depth of oracle $\mathcal {O}_1$ is about $2^8$ smaller than $\mathcal {O}_2$, and instead, $\mathcal {O}_2$ uses only one-seventh of the number of qubits required for $\mathcal {O}_1$. Both circuits consisting of $\mathcal {O}_1$ and $\mathcal {O}_2$ found the kernel with an accuracy greater than 86% probability in toy example simulations. Also, we analyzed the complexities of our Q-rMinRank attacks. The complexity of the Q-rMinRank attack is less than the complexity of the MinRank attacks with Grover’s algorithm estimated by the Rainbow team by $2^{30}$ in parameter set I, $2^{171}$ in parameter set III, and $2^{268}$ in parameter set V. Then, we estimated the quantum resources required for the Q-rMinRank attack. Our Q-rMinRank-Grover circuits consisting of oracle $\mathcal {O}_2$ require only 289, 833, and 1089 qubits for parameter sets I, III, and V of Rainbow, respectively. We also measured the quantum complexity by using the G-cost estimation, a metric for analyzing the security against quantum attacks on PQC. The quantum complexities of the Q-rMinRank-Grover algorithms are $2^{93}$, $2^{158}$, and $2^{174}$ for parameter sets I, III, and V, respectively, when applying oracle $\mathcal {O}_1$.

### Supplementary Information

Supplementary Information.

## Data Availability

All data generated or analyzed during this study are included in this published article.
