# Supplementary material for: Quantum rectangular MinRank attack on multi-layer UOV signature schemes
Source: Sci Rep. 2024 Jul 16;14:16340. doi: 10.1038/s41598-024-66841-0 (PMC11252166; doi:10.1038/s41598-024-66841-0)
Supplement: Supplementary file 1 — Supplementary Information. [file 41598_2024_66841_MOESM1_ESM.pdf]

A Quantum rectangular MinRank attack on HiMQ

HiMQ[1] is a multivariate quadratic-based digital signature algorithm that was standardized by the Korean Telecommunications Technology Association (TTA Standard) on June 17, 2020. It is a successor to HiMQ-3, which was submitted to the NIST Post-Quantum Cryptography Standardization project. Unlike HiMQ-3, which has a three-layer structure, HiMQ has a two-layer structure similar to Rainbow. Apart from the fact that the central map in HiMQ's private key has a sparse matrix structure, the overall key generation, signature generation, and verification processes are similar to those in Rainbow.

A.1 Algorithm Specification

- **Parameters**
  HiMQ is based on the hardness of the multivariate quadratic problem over a finite field $\mathbb{F}_q$ of size 2. The multivariate quadratic problem consists of a total of n variables (v vinegar variables and $o_1 + o_2$ oil variables, $n = v + o_1 + o_2$) and $m(= o_1 + o_2)$ equations. The recommended parameter sets for HiMQ based on different security strengths are shown in Table S1.

Parameter sets	q	v	o_1	o_2	λ
HiMQ-128	2^8	37	21	24	128
HiMQ-160	2^8	66	35	33	160
HiMQ-192	2^9	68	37	35	192

Table S1. The recommended parameter sets of HiMQ (λ: the security length)

- **Key Generation**
  The private keys of HiMQ consist of the inverse of the affine maps $S : \mathbb{F}_q^m \to \mathbb{F}_q^m$ and $T : \mathbb{F}_q^n \to \mathbb{F}_q^n$, namely S^{-1} and T^{-1}, and the central map $F = (F^{(1)}, ..., F^{(m)}) : \mathbb{F}_q^n \to \mathbb{F}_q^m$ composed of multivariate quadratic polynomials. The private keys are randomly selected using a pseudorandom function, and the public key $P = S \circ F \circ T$ is generated through the composition of the private keys.
  The m multivariate quadratic polynomials $(F^{(1)}, ..., F^{(m)})$, which constitute the central maps F, have n variables $x_1, ..., x_n$, and are generated as follows:

$$
\begin{aligned}
F^{(1)}(x_1,...,x_n) &= \Phi_1(x_1,...,x_v) + \delta_1 x_{v+1} x_{v+2} \\
F^{(2)}(x_1,...,x_n) &= \Phi_2(x_1,...,x_v) + \delta_2 x_{v+2} x_{v+3} \\
&\vdots \\
F^{(o_1)}(x_1,...,x_n) &= \Phi_{o_1}(x_1,...,x_v) + \delta_{o_1} x_{v+o_1} x_{v+1}
\end{aligned}
\tag{1}
$$

$$\Phi_i = \sum_{1 \le i < j}^{v} \alpha_{i,j} x_i x_j \tag{2}$$

$$
\begin{aligned}
F^{(o_1+1)}(x_1,...,x_n) &= \Psi_1(x_1,...,x_{v+o_1}) + \Theta_1(x_1,...,x_n) + \varepsilon_1 x_{o_1+1} + c_1 \\
&\vdots \\
F^{(o_1+o_2)}(x_1,...,x_n) &= \Psi_{o_2}(x_1,...,x_{v+o_1}) + \Theta_{o_2}(x_1,...,x_n) + \varepsilon_{o_2} x_{o_1+o_2} + c_{o_2}
\end{aligned}
\tag{3}
$$

$$\Psi_i = \sum_{j=1}^{v+o_1} \beta_{i,j} x_j x_{(i+j-1)(\bmod v+o_1)+1} \tag{4}$$

$$\Theta_i = \sum_{j=1}^{v+o_1} \gamma_{i,j} x_j x_{v+o_1+1+(j-i)(\bmod o_2)} \tag{5}$$

- **Signature Generation**
  Given a message M to be signed and a random r, the signature $\tau = (\sigma, r)$ is generated as follows:

- Compute $\zeta = (\zeta_1, ..., \zeta_m) = S^{-1}(H(M, r))$.
- Choose a random vector $s_v = (s_1, ..., s_v) \in \mathbb{F}_q^v$ and substitute to $x_1, ..., x_v$ of each quadratic polynomial $F^(i)$ $(1 \le i \le o_1)$.
- Find a solution $(x_{v+1}, ..., x_{v+o_1}) = (s_{v+1}, ..., s_{v+o_1})$ of system of quadratic equations $\begin{cases} \delta_1 x_{v+1} x_{v+2} = \zeta_1 - \Phi_1(s_v) \\ \vdots \\ \delta_{o_1} x_{v+o_1} x_{v+1} = \zeta_{o_1} - \Phi_{o_1}(s_v) \end{cases}$.
- Substitute $(s_1, ..., s_{v+o_1})$ to each $F^{(i)}$ and get a system of o_2 linear equations in o_2 variables.
- Find a solution $(s_{v+o_1+1}, ..., s_n)$ of the system of linear equations using Gaussian elimination.
- Compute $\sigma = (\sigma_1, ..., \sigma_n) = T^{-1}(s) \in \mathbb{F}_q^n$ for $s = (s_1, ..., s_n)$ that satisfies $F(s) = \zeta$.
- Output the $\tau = (\sigma, r)$ as the signature of message M.

- **Signature Verification**
  Given a message M, a signature $\tau = (\sigma, r)$, and a public key $P = (P_1, ..., P_m)$, the signature verification process is as follows:

  - Compute the hash value $H(M, r)$
  - Compute $P(\sigma) = (P_1(\sigma), ..., P_m(\sigma))$
  - If $P(\sigma) = H(M, r)$, the signature verification success

A.2 Applying Quantum rectangular MinRank attack to HiMQ

Our attack described in the "Quantum Rectangular MinRank Attack" section can be applied to HiMQ in a similar manner because the key structure of HiMQ is similar to that of Rainbow. The central map of HiMQ consists of sparse matrices, which leads to slight modifications in our attack.

A.2.1 The preprocessing step

HiMQ also has m public key matrices $P_1, \cdots, P_m$ with n rows and n columns. The public keys $P_1, \cdots, P_m$ are also converted into their polar form according to equations 3, 4, and 5. Due to the sparsity of the central map in HiMQ, the polar form conversion results in a sparse distribution of the secret values as well. The polar conversion of HiMQ and the distribution of the secret values are shown in Figure S1.

The matrices R_i $(1 \le i \le n)$ converted to polar form have non-zero secret coefficients of the first layer only in the $(v_1 + o_1)$-by-o_1 part in R_1 to $R_{v_1+o_1}$. After the polar form conversion, the linear combination M is computed in the *preprocessing* step according to equation 6, and the $(v_1 + o_1)$-by-o_1 part of M (as in equation 7) is transferred to the *quantum kernel extraction* step.

A.2.2 The quantum kernel extraction step

A Q-rMinRank-Grover algorithm is used to find a kernel of the matrix M' with v_1 rows and o_1 columns in the *quantum kernel extraction* step. The overall process of the *quantum kernel extraction* step is similar to Algorithm 4. The difference between the Q-rMinRank-Grover algorithm for Rainbow and HiMQ lies in the size of the input matrix M' and the number of non-zero elements. The Q-rMinRank-Grover algorithm finds the kernel of v_1-by-o_1 matrix M', which has $v_1 \cdot o_1$ non-zero elements in the case of Rainbow, and of $(v_1 + o_1)$-by-o_1 matrix M', which has $2o_1 + (v_1 + o_1) \cdot o_2$ non-zero elements.

A.2.3 The key recovery step

In the *key recovery* step, the kernel vector Γ obtained from the *quantum kernel extraction* step is utilized to compute the linear combination of the public keys, as shown in Equation 8. By computing this linear combination, the central map of HiMQ can be recovered as in "The Key Recovery Step" section, allowing for the recovery of the secret key.

A.2.4 Quantum Resource Analysis

Unlike Rainbow, where the input matrix has a size of v_1-by-o_1, the input matrix for the Q-rMinRank-Grover algorithm for HiMQ attack is $(v_1 + o_1)$-by-o_1. Since the central map of HiMQ is sparse, not all elements need to be multiplied by quantum multipliers. To carry out the quantum rectangular MinRank attack on HiMQ, we designed two oracle circuits for the Q-rMinRank-Grover algorithm, considering both depth and width, similar to the approach used for the Rainbow case. The concept of oracle circuits, excluding the number of quantum multipliers and adders, is similar to that presented in the "Oracle Circuit Designs" section.

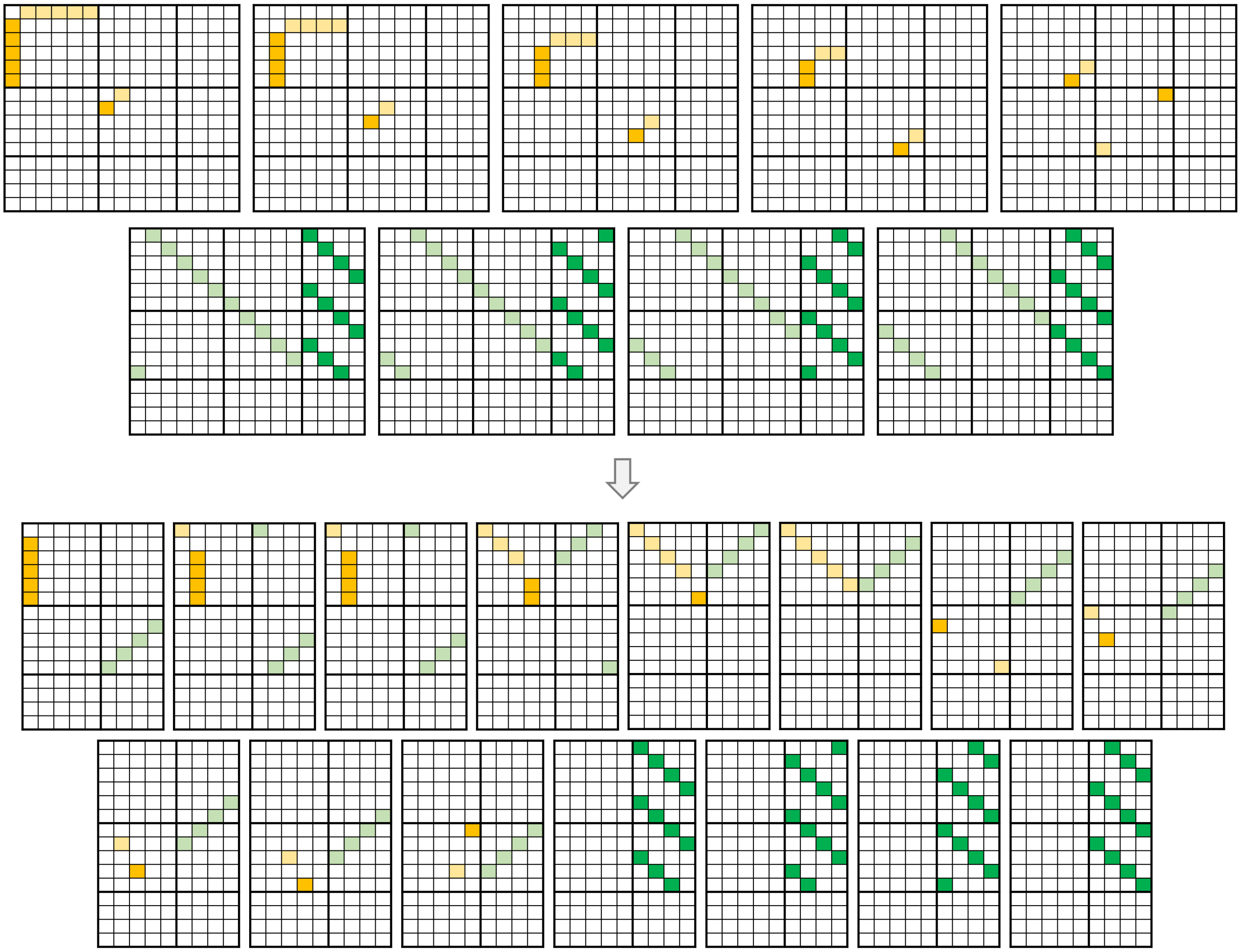

Figure S1. The polar form conversion of HiMQ ($v = 6$, $o_1 = 5$, $o_2 = 4$)

Like in the "Quantum Resource Analysis" section, we estimated the quantum resources (#QB, G-cost, and D) required for the Q-rMinRank-Grover algorithm used in HiMQ key recovery. Table S2 shows the quantum resources for our Q-rMinRank_Oracle and Diffusion pair for HiMQ.

Compared with Rainbow parameter sets with the same security level, we observed that our quantum circuits for HiMQ attacks require a larger number of qubits, but the G-cost and D are somewhat smaller. To calculate the total quantum complexity of our quantum attack, we need to multiply the quantum resource of the oracle and diffusion pair from Table S2 by the number of Grover iterations obtained from Equation 9. Table S3 shows the G-cost by MAXDEPTH and the quantum complexity of Q-rMinRank-Grover for HiMQ.

HiMQ uses a larger finite field than Rainbow parameter sets with the same security level. This means allocating the kernel vector elements to quantum registers requires more qubits, increasing Grover iterations. As a result, despite the sparsity of HiMQ's central map as a sparse matrix, it still exhibits a higher quantum complexity than Rainbow's quantum circuit. According to our analysis, HiMQ-128 does not satisfy security level I (quantum complexity greater than 2^{157}/MAXDEPTH), and HiMQ-160 and HiMQ-192 do not meet security level III (quantum complexity greater than 2^{221}/MAXDEPTH).

References

1. Association, K. T. T. Post quantum cryptography based on multivariate quadratic equations - part 2: Himq, digital signature algorithm with appendix. *TTAK.KO-12.0348-Part2* (2020).

Algorithm	Parameter sets	Oracle	#QB	G-cost	D	DW	D^2W
HiMQ	128	$\mathcal{O}_1$	3662	1.54×2^{19}	1.93×2^{10}	$2^{22.8}$	$2^{33.7}$
		$\mathcal{O}_2$	665	1.50×2^{20}	1.79×2^{17}	$2^{27.2}$	$2^{45.1}$
	160	$\mathcal{O}_1$	6459	1.13×2^{21}	1.61×2^{11}	$2^{24.3}$	$2^{36.0}$
		$\mathcal{O}_2$	1121	1.10×2^{22}	1.31×2^{19}	$2^{29.5}$	$2^{48.9}$
	192	$\mathcal{O}_1$	7036	1.22×2^{21}	1.70×2^{11}	$2^{24.5}$	$2^{36.3}$
		$\mathcal{O}_2$	1312	1.18×2^{22}	1.42×2^{19}	$2^{29.9}$	$2^{49.4}$

G-cost: the number of quantum gates used in an oracle and diffusion pair
D: the depth for an oracle and diffusion pair
DW: the depth times width for an oracle and diffusion pair (serial overhead)
D^2W: the square of depth times width for an oracle and diffusion pair (parallel overhead)

Table S2. The quantum resources for our Q-rMinRank_Oracle and Diffusion pair

Algorithm	Parameter sets	Oracle	MD	G-cost	D	$Complexity_{quantum}$
HiMQ	128	$\mathcal{O}_1$	2^{40}	1.48×2^{73}	1.00×2^{40}	1.48×2^{113} / MAXDEPTH
			2^{64}	1.48×2^{49}	1.00×2^{64}	
			2^{96}	1.48×2^{17}	1.00×2^{96}	
		$\mathcal{O}_2$	2^{40}	1.34×2^{81}	1.00×2^{40}	1.34×2^{121} / MAXDEPTH
			2^{64}	1.34×2^{57}	1.00×2^{64}	
			2^{96}	1.34×2^{25}	1.00×2^{96}	
	160	$\mathcal{O}_1$	2^{40}	1.81×2^{131}	1.00×2^{40}	1.81×2^{171} / MAXDEPTH
			2^{64}	1.81×2^{107}	1.00×2^{64}	
			2^{96}	1.81×2^{75}	1.00×2^{96}	
		$\mathcal{O}_2$	2^{40}	1.44×2^{140}	1.00×2^{40}	1.44×2^{180} / MAXDEPTH
			2^{64}	1.81×2^{107}	1.00×2^{64}	
			2^{96}	1.81×2^{75}	1.00×2^{96}	
	192	$\mathcal{O}_1$	2^{40}	1.03×2^{159}	1.00×2^{40}	1.03×2^{199} / MAXDEPTH
			2^{64}	1.03×2^{135}	1.00×2^{64}	
			2^{96}	1.03×2^{103}	1.00×2^{96}	
		$\mathcal{O}_2$	2^{40}	1.68×2^{167}	1.00×2^{40}	1.68×2^{207} / MAXDEPTH
			2^{64}	1.68×2^{143}	1.00×2^{64}	
			2^{96}	1.68×2^{111}	1.00×2^{96}	

Table S3. The G-cost by MAXDEPTH and the quantum complexity of Q-rMinRank-Grover for HiMQ
